# Evolutionary origin and functional divergence of totipotent cell homeobox genes in eutherian mammals

**DOI:** 10.1186/s12915-016-0267-0

**Published:** 2016-06-13

**Authors:** Ignacio Maeso, Thomas L. Dunwell, Chris D. R. Wyatt, Ferdinand Marlétaz, Borbála Vető, Juan A. Bernal, Shan Quah, Manuel Irimia, Peter W. H. Holland

**Affiliations:** Department of Zoology, University of Oxford, South Parks Road, Oxford, OX1 3PS UK; Centro Andaluz de Biología del Desarrollo, Consejo Superior de Investigaciones Científicas/Universidad Pablo de Olavide, 41013 Sevilla, Spain; Centre for Genomic Regulation, Barcelona Institute of Science and Technology, Dr. Aiguader 88, 08003 Barcelona, Spain; Universitat Pompeu Fabra (UPF), Barcelona, Spain; Institute of Enzymology, Research Centre for Natural Sciences, Hungarian Academy of Sciences, Budapest, Hungary; Centro Nacional de Investigaciones Cardiovasculares (CNIC), Madrid, Spain

**Keywords:** Tandem duplication, Asymmetric evolution, Embryo, Placental, Homeodomain, PRD

## Abstract

**Background:**

A central goal of evolutionary biology is to link genomic change to phenotypic evolution. The origin of new transcription factors is a special case of genomic evolution since it brings opportunities for novel regulatory interactions and potentially the emergence of new biological properties.

**Results:**

We demonstrate that a group of four homeobox gene families (*Argfx*, *Leutx*, *Dprx*, *Tprx*), plus a gene newly described here (*Pargfx*), arose by tandem gene duplication from the retinal-expressed *Crx* gene, followed by asymmetric sequence evolution. We show these genes arose as part of repeated gene gain and loss events on a dynamic chromosomal region in the stem lineage of placental mammals, on the forerunner of human chromosome 19. The human orthologues of these genes are expressed specifically in early embryo totipotent cells, peaking from 8-cell to morula, prior to cell fate restrictions; cow orthologues have similar expression. To examine biological roles, we used ectopic gene expression in cultured human cells followed by high-throughput RNA-seq and uncovered extensive transcriptional remodelling driven by three of the genes. Comparison to transcriptional profiles of early human embryos suggest roles in activating and repressing a set of developmentally-important genes that spike at 8-cell to morula, rather than a general role in genome activation.

**Conclusions:**

We conclude that a dynamic chromosome region spawned a set of evolutionarily new homeobox genes, the ETCHbox genes, specifically in eutherian mammals. After these genes diverged from the parental *Crx* gene, we argue they were recruited for roles in the preimplantation embryo including activation of genes at the 8-cell stage and repression after morula. We propose these new homeobox gene roles permitted fine-tuning of cell fate decisions necessary for specification and function of embryonic and extra-embryonic tissues utilised in mammalian development and pregnancy.

**Electronic supplementary material:**

The online version of this article (doi:10.1186/s12915-016-0267-0) contains supplementary material, which is available to authorized users.

## Background

Homeobox genes encode transcription factors with a recognisable DNA-binding domain, the homeodomain, and most play regulatory roles in cell fate determination and embryonic patterning in animals. The majority of metazoan homeobox genes, including those of human, are members of small gene families that have been highly conserved through the evolutionary diversification of the bilaterians [1]. These arose by extensive homeobox gene duplication and divergence early in animal evolution, generating around 100 conserved gene families, and there has been little further elaboration apart from expansion due to genome duplication in vertebrates [2, 3]. A small number of lineage-specific tandem gene duplications have occurred, and these raise questions concerning how evolutionarily young homeobox genes are recruited to new regulatory roles. For example, divergent tandem duplicates of the *Hox3* gene have been recruited for extra-embryonic membrane specification and patterning in dipteran and lepidopteran insects [4, 5], a large expansion of the Rhox homeobox gene family is deployed in reproductive tissues of mouse [6], and duplicates of TALE class genes are expressed in early development of molluscs [7]. This theme of recruitment of novel genes to roles in early development or reproduction is compatible with the ‘hourglass’ or ‘phylotypic stage’ model of developmental evolution, which postulates that early (and late) stages of embryonic development are most prone to evolutionary modification [7–9]. However, with the exception of the *Hox3* duplicates, the evolutionary origin of most ‘lineage-specific’ homeobox genes is unknown.

A small number of divergent PRD class homeobox genes have very restricted phylogenetic distribution and offer an opportunity to examine the evolutionary origin and functional recruitment of novel homeobox genes in mammals. *ARGFX*, *DPRX*, *LEUTX* and two *TPRX* genes were originally identified in the human genome [1, 10]. Orthologous sequences were later identified from some other eutherian (placental) mammals, excluding mouse and rat [11, 12], although for *Argfx*, the coding sequence is frequently disrupted by disabling mutations [12]. It has been proposed that these genes arose by tandem duplication from the *Crx* gene, a member of the Otx gene family [13], although this hypothesis has only been tested for *Argfx* [10, 12]. Furthermore, the deduced protein sequences are extremely divergent from *Crx* implying that, if this hypothesis is correct, the homeodomains must have undergone extensive sequence divergence after duplication, together with transposition along a chromosome (for *Dprx* and *Leutx*) or to another chromosome (for *Argfx*). Such a scenario would imply ‘asymmetric evolution’ whereby functional constraints compel a ‘parental’ gene (*Crx*) to retain ancestral sequence and functions, while ‘daughter’ genes can diverge until a new function is acquired [14].

The proposed parental gene, *Crx* (*Cone-rod homeobox*), is expressed primarily in photoreceptors of the vertebrate eye in mammals, a feature not shared by the putative daughter genes. Instead, evidence for trace expression has been reported in testis and human embryonic stem cells (hESCs) for human *ARGFX* [10, 11] and testis for human *TPRX1* [10]. By far the clearest evidence of strong expression for each of the genes is in early human embryos, around the 4-cell, 8-cell, and morula stages [15–17]. At the morula stage all cells are equivalent in terms of developmental potential, fulfilling the principal definition of totipotency – that each cell may contribute to any descendent cellular lineage of embryonic or extra-embryonic tissues [18, 19]. It is not clear if human 8-cell stage cells are totipotent in the stricter sense of an individual cell being capable of constituting a viable embryo, a property which may exist only until the 4-cell stage due to diminishing cell size [18]. Evidence from other mammals suggests that cell fate becomes gradually restricted after blastocyst formation, although totipotency may be retained by some inner cell mass of early blastocysts [19–21]. hESCs are thought to mimic later developmental stages, such as pluripotent stem cells of the epiblast [19].

These findings raise the intriguing possibility that a set of evolutionarily new homeobox genes arose sometime in mammalian evolution and, after extensive sequence divergence, were recruited to regulatory roles in totipotent embryonic cells. Here, we test this evolutionary scenario using comparative genomic analyses and functional assays. We demonstrate that *Argfx*, *Dprx*, *Leutx* and *Tprx* genes arose by asymmetric evolution from the *Crx* homeobox gene at the base of eutherian evolution, as part of a dynamic series of duplications that also yielded a novel gene (*Pargfx*) and other genes that have been lost. Transfection experiments provide evidence that human ARGFX, DPRX, LEUTX and TPRX1 proteins localise to the nucleus, and that *ARGFX*, *LEUTX* and *TPRX1* regulate a set of developmentally-important genes precisely modulated at the time when the earliest cell fate decisions are made.

## Results

### Dynamic gain and loss reveals an unstable genomic region

**Fig. 1:**
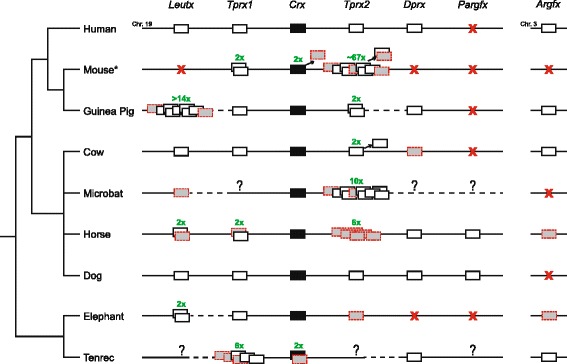
Presence, absence and duplication of *Crx* and *Crx*-derived homeobox genes in placental mammals. The order of relevant genes on human chromosomes 19 and 3 is compared to orthologues in eight other mammals; grey boxes with dashed outline indicate probable pseudogenes, X indicates gene deletion, question marks indicate missing data, dotted lines show unconnected scaffolds. The asterisk in mouse indicates that, in this species, *Tprx1* and *Tprx2* orthologues are especially divergent and named *Crxos* and *Obox* genes, respectively. Many intervening non-homeobox genes are not shown. Human, mouse and guinea pig belong to Euarchontoglires, cow, bat, horse and dog to Laurasiatheria; together, these clades form Boreoeutheria. Elephant and tenrec are Afrotheria within the Atlantogenata

We found orthologues of *Argfx*, *Dprx*, *Leutx*, *Tprx1* and *Tprx2* in diverse species from the main eutherian branches – Boreoeutheria (comprising Euarchontoglires and Laurasiatheria) and Atlantogenata (comprising Afrotheria and Xenarthra) – but not in monotremes or marsupials (Fig. 1). In human, *Tprx1* and *Tprx2* flank the *Crx* gene on chromosome 19, *Dprx* and *Leutx* are more distant on chromosome 19, and *Argfx* is on chromosome 3. In each eutherian mammal genome examined, the genes are located in syntenic positions defining orthology relationships (Additional file 1: Figure S1). We also uncover a previously undescribed locus we term *Pargfx* (*Parent of Argfx*) in several carnivores (dog, cat and ferret) and odd-toed ungulates (rhinoceros and horse), adjacent to *Dprx* (Fig. 1; Additional file 1: Figure S1; Additional file 2: Figure S2); a retroposed copy is present in primate genomes. These data indicate that *Argfx*, *Dprx*, *Leutx*, *Pargfx* and *Tprx* are eutherian-specific homeobox gene families that originated after the divergence of the placental and marsupial lineages.

**Fig. 2:**
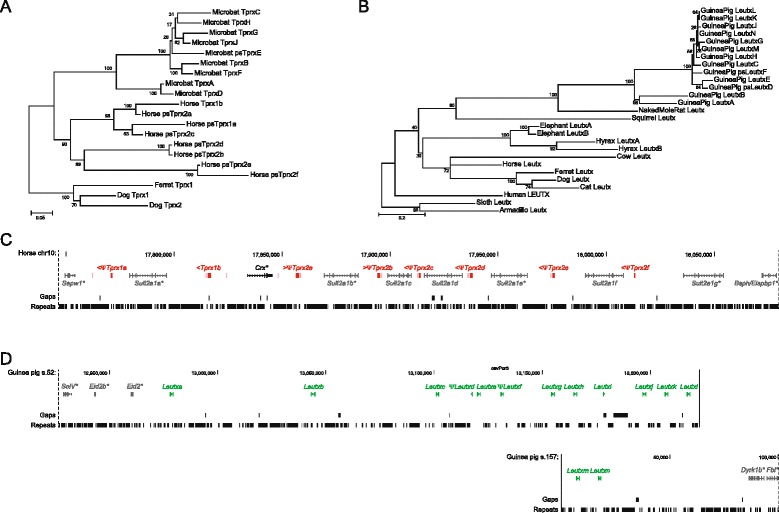
Additional duplication of *Tprx* and *Leutx*. **a** Maximum likelihood phylogenetic tree of duplicate horse and bat *Tprx* loci rooted with ferret and dog *Tprx* genes. **b** Maximum likelihood phylogenetic tree of duplicate guinea pig, elephant and hyrax *Leutx* genes with a range of other mammalian *Leutx* genes. **c** Genomic organisation of duplicated horse *Tprx* loci, the majority of which are pseudogenes. **d** Genomic organisation of duplicated guinea pig *Leutx* loci

Lability did not cease after divergence of the major mammalian lineages. We detect examples of additional gene gain, including an extra *Leutx* in elephant and hyrax, 14 *Leutx* copies in the guinea pig genome, at least 8 *Tprx* duplicates in horse and 10 in bat, and an extra *Tprx* gene shared by cows and pigs (Figs. 1 and 2; Additional file 3: Table S1). The cow/pig *Tprx3* locus is unusual in being located between 4.5 and 8 Mb away from *Tprx1* and *Tprx2*, within the leukocyte receptor complex – a cluster of immunoglobulin-like receptor genes [22].

We also detect many cases of gene inactivation and loss. All species examined have lost one or more of the eutherian-specific homeobox gene families; for example, humans have lost *Pargfx*. For both *Dprx* and *Pargfx*, degeneration has occurred repeatedly on at least three occasions within mammals (Fig. 1; Additional file 3: Table S1). The most extreme loss is seen in mouse and rat, where *Argfx*, *Dprx*, *Leutx* and *Pargfx* are all missing.

*Tprx1* and *Trpx2* are not readily found in mouse and rat, but in this case, our analyses reveal the explanation is not gene loss but extensive sequence divergence, contrary to previous reports [12, 23]. The mouse genome has a highly divergent double-homeobox gene *Crxos* in the position syntenic to *Tprx1*, and a massive expansion of approximately 67 tandem loci (including pseudogenes) of the murid-specific *Obox* genes in the position syntenic to *Tprx2*. The extreme sequence differences between *Crxos* and *Tprx1*, and between *Obox* genes and *Tprx2*, led to the proposal that *Crxos* and *Obox* genes arose independently in rodents, after loss of *Tprx1* and *Tprx2* [12, 23]. To test if *Crxos* and *Obox* loci are cryptic orthologues of *Tprx* genes, we examined genomes of other rodent species. We found that squirrels and ctenohystricans (naked mole-rat and guinea pig) have a complement of PRD class genes similar to non-rodent eutherian mammals. Furthermore, inclusion of genes from guinea pig and naked mole-rat into phylogenetic analysis broke the long branches to *Crxos* and *Obox* genes, and gave high support for their orthology with *Tprx1* and *Tprx2*, respectively (Additional file 4: Figure S3). In several species, evolutionary relationships are further disguised by apparent gene conversion between *Tprx1* and *Tprx2*.

The generation of a new set of homeobox genes in eutherian mammals is highly unusual. The continued duplication of these genes, mirrored by extensive and recurrent gene loss, combine to reveal an exceptionally dynamic region of the eutherian genome (syntenic to part of human chromosome 19), expanding and contracting to spawn and delete new homeobox genes at a high rate.

### *Crx* gave rise to* Argfx*, *Dprx*, *Leutx*, *Pargfx *and *Tprx *by asymmetric evolution

**Fig. 3:**
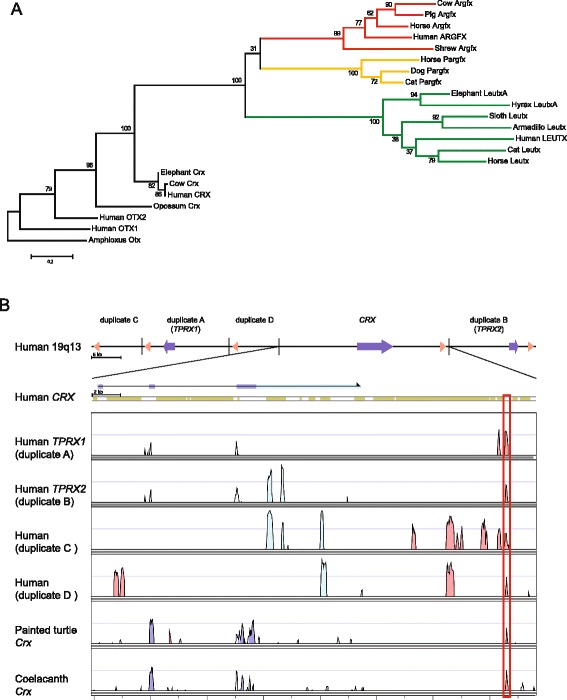
Evidence for origin of *Argfx*, *Pargfx*, *Leutx* and *Tprx* genes from *Crx*. **a** Maximum likelihood phylogenetic tree using homeodomains plus C-terminal Otx-specific domain. **b** Position of conserved non-coding elements (CNEs) depicted as red arrowheads, located 3’ to human *CRX*, *TPRX1* and *TPRX2*, plus two ghost loci (duplicates C and D) inferred to have lost coding sequences. Sequence identity between the CNEs (boxed) is shown by the VISTA plot, which also reveals a single location of the same ancient CNE next to *Crx* in turtle and coelacanth. VISTA peaks: blue, coding; turquoise, untranslated region; pink, noncoding. Masked repetitive sequences are indicated by khaki segments above the VISTA plot

Analysis using homeodomain sequences does not unambiguously reveal the progenitor gene for *Argfx*, *Dprx*, *Leutx*, *Pargfx* and *Tprx* genes from within the PRD class [1]. However, we discovered that we could expand the length of phylogenetically informative sequence by including amino acid stretches C-terminal to the homeodomain conserved between Argfx, Leutx, Pargfx and Otx proteins, corresponding to the Otx-specific domain [24] (Additional file 2: Figure S2). Phylogenetic analysis using this alignable region plus the homeodomain (Additional file 4: Figure S3) revealed that the eutherian *Crx* gene is the sister (and progenitor) to *Argfx*, *Leutx* and *Pargfx* (Fig. 3a). Although we did not detect the same motif in *Dprx* or *Tprx* genes, the tight physical linkage to *Pargfx* or *Crx*, respectively, plus the PRD class homeodomain assignment make it highly likely that these genes are also divergent cryptic paralogues of *Crx*. Since duplicated loci can sometimes retain similar conserved non-coding elements (CNEs) [25, 26], we also turned to non-coding DNA for additional evidence of ancestry. We divided the human *TPRX1*-*CRX*-*TPRX2* genomic region into fragments and performed VISTA comparisons between them using *CRX* as a reference (Fig. 3b). This revealed five copies of a duplicated CNE, all of which could also be detected in other placental mammal species; non-eutherian species contain only a single copy of the CNE adjacent to *Crx* (Fig. 3b; Additional file 2: Figure S2). Of the five copies in human, one is located downstream to each of *TPRX1*, *TPRX2* and *CRX*, in the same relative orientation, giving strong evidence that *Tprx* genes are derived from *Crx* by tandem duplication and asymmetric divergence (Fig. 3b). Interestingly, the additional two CNE copies are more distant and not associated with homeobox loci, and are most likely remnants of two further ‘ghost’ *Crx* tandem duplicates, lost early in mammalian evolution as part of the dynamic evolution elucidated above.

### Functional constraints in divergent homeodomains

**Fig. 4:**
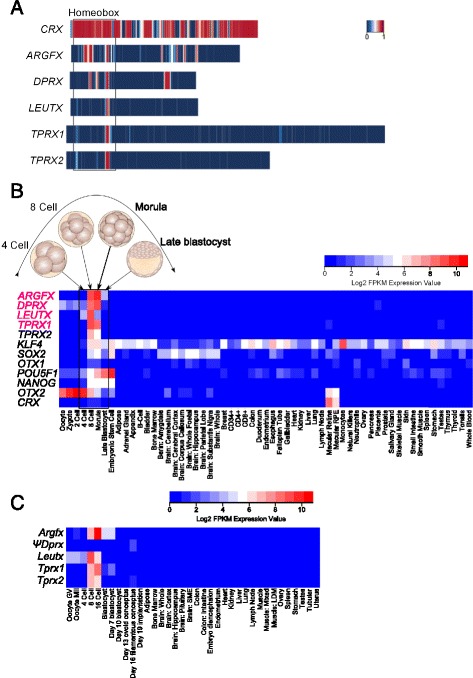
Evidence for function from sequence and expression. **a** Heatmaps showing placental mammal phastCons conservation probability scores for the coding sequences of human *CRX* and ETCHbox genes; from 0 (blue) to 1 (red). The most consistent conserved (red) stretch codes for homeodomain helix 3. **b** Heatmaps showing expression profiles in developmental stages and adult tissues of human ETCHbox genes compared to paralogues *OTX2* and *CRX*, and stem cell markers, according to fragments per kilobase per million reads (FPKM) on a log2 scale (red, high expression; blue, low expression). **c** Heatmaps showing expression profiles in developmental stages and adult tissues of cow ETCHbox genes, according to FPKM on a log2 scale (red, high expression; blue, low expression)

The extreme sequence divergence of *Argfx*, *Dprx*, *Leutx*, *Pargfx* and *Tprx* gene from their highly conserved progenitor, *Crx*, together with the prevalence of gene loss and pseudogenes, raises questions about whether they are functional. We also note that sequence differences between species, inferred by phylogenetic branch lengths, are higher than for other homeobox genes (Fig. 3a; Additional file 4: Figure S3). To assess this quantitatively, we used phastCons, a phylogenetic hidden Markov model-based method that estimates the probability that each nucleotide is part of a conserved element, regardless of coding potential [27]. Applied to *Leutx*, *Tprx1* and *Tprx2*, this identified just 13 to 19 nucleotide positions with > 50 % probability of belonging to a conserved element; *Argfx* and *Dprx* showed more apparent conservation with 95 and 279 positions. This contrasts to over 600 positions for the progenitor gene *Crx*. Nonetheless, for each gene, the highest similarity and conservation probability between eutherian species is consistently the third alpha helix of the homeodomain, suggestive of selective pressures to retain DNA-binding (Fig. 4a).

Specificity of expression can also be a clue to functionality. To assess this, we first built improved gene models for human *ARGFX*, *DPRX*, *LEUTX*, *TPRX1* and *TPRX2*, refining intron/exon boundaries using sequence alignment and transcriptome data (Additional file 2: Figure S2). After integrating these into a human reference gene dataset, we mapped available RNAseq reads from human tissues and developmental stages to the human genome, and plotted heatmaps of expression (Fig. 4b). This revealed tightly regulated temporal expression profiles for each gene restricted to pre-blastocyst stages. *ARGFX* and *TPRX1* are activated with embryonic genome activation at the 8-cell stage, with lower level *DPRX* and *LEUTX* transcription initiated slightly earlier. *TPRX2* has the lowest expression but mirroring *TPRX1*. The expression level for all five genes (*ARGFX*, *DPRX*, *LEUTX*, *TPRX1* and *TPRX2*) crashes after the morula stage, and the genes are not strongly expressed again in embryonic, foetal or adult tissues. These expression profiles are tighter and developmentally earlier than the classical pluripotent stem cell markers *POU5F1*, *NANOG*, *KLF4* and *SOX2*, which are expressed in the blastocyst and in hESCs (Fig. 4b). The expression profiles are also different to the human orthologue of the progenitor gene, *CRX*. To examine if the expression patterns differ between species, we also analysed RNAseq data from cow embryos and adult tissues [28, 29]. This revealed that bovine *Leutx*, *Tprx1*, *Tprx2* and *Argfx* gene expression peaks sharply at the 8-cell to 16-cell stages, very similar to expression of their human orthologues (Fig. 4c). *Dprx* is a pseudogene in cow. As noted above, the orthologues of *Tprx* genes are extensively duplicated and highly divergent in mice, but even so some of these are expressed primarily between the 2-cell or 4-cell stage and morula [30, 31], with transcripts from some loci in oocytes, later embryogenesis and placenta [32, 33]. The finding that the *Argfx*, *Dprx*, *Leutx* and *Tprx* genes are phylogenetically related to each other, eutherian-specific and expressed primarily in pre-blastocyst totipotent stages of human, cow and mouse development suggests a collective name is helpful. We denote these genes, plus *Pargfx*, the ETCHbox (Eutherian Totipotent Cell Homeobox) genes.

### Ectopic expression of ETCHbox genes reveals differential transcriptional activities

**Fig. 5:**
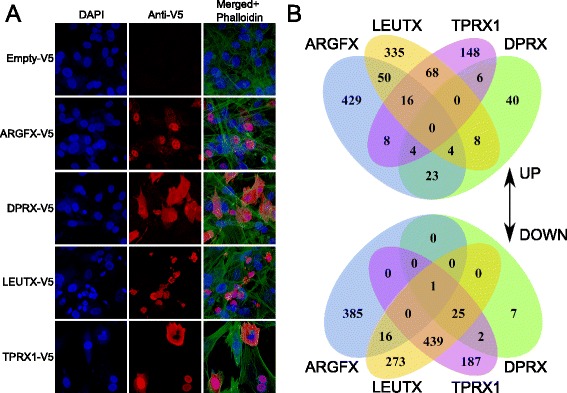
Nuclear localisation and transcriptional activity. **a** Confocal images of fibroblasts transfected with V5-tagged constructs or empty vector control, showing nuclei (blue DAPI), ectopic protein (red) and, in the merged images, actin cytoskeleton (green phalloidin). **b** Numbers of genes up- and down-regulated by ectopic expression and overlaps between responsive gene sets. The clearest overlap is between *TPRX1* and *LEUTX* down-regulated genes. For stringency criteria, see Methods

Since the human embryonic stages expressing ETCHbox genes are not readily amenable to experimental analysis, we deployed ectopic expression to gain insight into functions. The mouse system is not appropriate since mouse has lost most ETCHbox genes while extensively duplicating *Tprx* genes. Based on the refined gene models described above, we used a combination of exon-specific genomic PCR and gene synthesis to build constitutive expression constructs for the four most highly-expressed human ETCHbox genes: *ARGFX*, *DPRX*, *LEUTX* and *TPRX1*. Reading frames were C-terminal tagged with V5 to facilitate protein detection, and constructs transfected into primary human fibroblasts. Western blot analysis confirmed that each construct generated a full-length protein (Additional file 5: Figure S4). Immunocytochemistry revealed all four proteins localise to the nucleus, but with subtly different subcellular patterns. ARGFX and TPRX1 proteins were clearly localised to the nucleus, apart from nucleoli; DPRX protein was strongest in the nucleus but also in cytoplasm; LEUTX was nuclear-localised but with evidence of disruption to cell integrity (Fig. 5a). The significance of the subtle differences is unclear; the predominant nuclear location is as expected for transcription factors. Nuclear localisation was also found using specific antibodies to ARGFX and DPRX proteins expressed in HeLa cells from untagged constructs (not shown).

We used transcriptome analysis to assess if ectopic expression in fibroblasts caused activation or inhibition of specific genes and genetic pathways. After culture for 48 h, RNAseq in triplicate samples was performed using the Illumina platform to detect up- and down-regulated genes and pathways compared to control transfected cells. Differential biological effects of each ETCHbox gene are evident when lists of significantly up- and down-regulated genes are compiled and compared between treatments, with *DPRX* expression causing a smaller change to the total transcriptome (Fig. 5b). This revealed between 1143 and 1839 genes significantly up- or down-regulated by *ARGFX*, *LEUTX* or *TPRX1*, of which 250–754 had a fold-change of > 1.25 (equivalent to 2-fold per transfected cell). There is very strong overlap between gene sets down-regulated by *LEUTX* and *TPRX1* (Fisher’s exact test *P* = 0) and significant overlap between their up-regulated genes (*P* = 10^–73^). *ARGFX* has a large number of gene-specific effects. These experiments verify transcriptional activity of the ETCHbox proteins and suggest partial overlap of function between *LEUTX* and *TPRX1*.

### Relevance of transcriptional activities to human embryonic development

To investigate if downstream genes detected in cell culture are functionally relevant to early human development, we deployed a temporal clustering approach. First, we determined the temporal expression profiles for all genes expressed between the oocyte and blastocyst stages of human development. A gene expression clustering approach identified 106 distinct expression profiles. Second, we tested whether any of these profiles are significantly enriched in genes up- or down-regulated by transfection of *ARGFX*, *LEUTX* or *TPRX1*, determined above. *DPRX* was not considered due to the smaller number of specific transcriptional effects elicited by transfection.

**Fig. 6:**
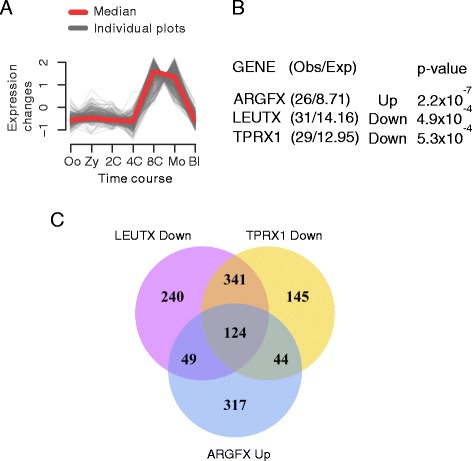
Association with human embryo gene expression and antagonism between genes. **a** Profile 27, out of 106 temporal profiles of human embryonic gene expression, is shown; grey lines are expression plots of 313 individual genes in the profile, red marks the median. Oo, oocyte; Zy, zygote; 2C, 2-cell; 4C, 4-cell; 8C, 8-cell; Mo, morula; Bl, blastocyst. **b** Genes up-regulated by ectopic *ARGFX*, or down-regulated by *LEUTX* or *TPRX1*, are enriched in profile 27. **c** Venn diagram showing strong overlap between *LEUTX* and *TPRX1*-down regulated genes and *ARGFX* up-regulated genes, demonstrating the antagonistic effect extends to the full transcriptional response induced by ectopic expression not just the subset in profile 27

We detect a strong and striking enrichment of *ARGFX*, *LEUTX* and *TPRX1* responsive genes within human expression profile 27 (Fig. 6a,b). This set of 50 human genes has low or zero expression in the oocyte, zygote, 2-cell and 4-cell stages, then a sharp transition to high expression at the 8-cell and morula stages, and a rapid drop in expression before the blastocyst stage; these genes therefore have a pulse of gene expression around genome activation and prior to cell fate determination. This profile differs from several others that show an increase of expression at genome activation but distinct patterns of subsequent expression. The only other signal detected is slight enrichment for *LEUTX* up-regulated genes in profile 40 (χ^2^*P* = 0.02). It is notable that, for *ARGFX*, it is the set of genes that are up-regulated by transfection in cultured cells that is strongly enriched within profile 27 (*P* = 2 × 10^–7^), while for *LEUTX* and *TPRX1* enrichment is detected for genes down-regulated by transfection (*P* = 5 × 10^–4^). Comparison of genes underlying the enrichment in each case reveals extensive overlap (Additional file 6: Figure S5). To examine if expression of putative targets is conserved in other mammals, we examined their bovine orthologues. The 50 human genes have 33 bovine orthologues with variable expression; clustering revealed five temporal profiles. Three profiles, containing 58 % of comparable genes (19/33), have expression profiles very similar to human profile 27, with a clear peak of expression at the 8-cell to 16-cell stages (Additional file 7: Figure S6).

The discovery that a set of genes in profile 27 responds to expression of human *LEUTX* and *TPRX1* in the opposite direction to their response to *ARGFX* prompted us to test if this relationship extends to the full dataset of responsive genes. Comparison of all human targets down-regulated by *TPRX1* and by *LEUTX* (not just those in profile 27) with all genes up-regulated by *ARGFX*, reveals over 100 target genes in common (Fig. 6c); *ARGFX*-up/*LEUTX-*down overlap *P* = 1.2 × 10^–116^, *ARGFX*-up/*TPRX1*-down *P* = 9.3 × 10^–122^ (Fisher’s exact test). No other combination of three datasets reveals a similar extent of overlap (Additional file 8: Figure S7). These analyses suggest that human *LEUTX* and *TPRX1* genes act on a similar set of genes and genetic pathways between the 4-cell stage and the blastocyst, in an antagonistic manner to the effects of *ARGFX*. In several cases, *ARGFX* is causing an increase in the transcription of genes that specifically rise sharply in expression before the 8-cell stage, whereas *LEUTX* and *TPRX1* down-regulate these same genes, which, in the embryo, drop dramatically in expression between morula and blastocyst. Together, we suggest that *LEUTX*, *TPRX1* and *ARGFX* transcription factors modulate the precise on/off ‘pulse’ of expression that characterises profile 27, at the time when the very first cell fate decisions are made in the early mammalian embryo, leading to specification of embryonic and extra-embryonic cell lineages.

## Discussion

A defining feature of therian mammals (marsupials and placentals) is internal retention of the developing embryo, involving implantation, protection and active nourishment through specialised extra-embryonic tissues. Monotremes, birds and reptiles also have extra-embryonic tissues but generally do not face the same challenges of prolonged maternal retention and implantation. Extra-embryonic membranes are especially sophisticated in placental mammals which retain the developing embryo for longer than do marsupials. Key to the development of extra-embryonic membranes are the very earliest cell fate decisions made in development [34]. After fertilization, a series of cell divisions produces a ball of identical cells, the morula. The end of the morula stage is marked by cellular compaction and the first cell fate decisions as a hollow blastocyst is generated, comprising an outer cell layer fated to become extraembryonic trophectoderm (which forms the majority of the foetal contribution to the placenta) around a multicellular inner cell mass. The inner cell mass rapidly undergoes a further cell fate decision to specify a second extraembryonic layer of cells (parietal endoderm which contributes to yolk sac) around the true embryonic cells or epiblast. The ETCHbox genes we analyse in this study, or at least their human and bovine orthologues, are expressed just prior to (and to a lesser extent during) these cell fate decisions that establish the critical distinctions between embryonic and extra-embryonic tissues of the mammalian embryo. This precise temporal pattern of gene expression suggests they are likely to have roles in the totipotent morula stage, when the necessary cellular conditions are established for cell fate specification, and subsequent mammalian development and pregnancy. The collective name we propose, ETCHbox genes, reflects their expression and evolutionary origin.

In seeking to determine the precise molecular roles of these genes, the biggest challenges are practical and ethical issues concerning experimental manipulation of the earliest developmental stages of mammals, particularly humans. Mice cannot be used as straightforward models since, as we demonstrate, murid rodents have lost *Argfx*, *Dprx*, *Leutx* and *Pargfx*, and have undergone duplication and further radical sequence divergence of *Tprx* genes. Lack of expression after blastocyst stage also precludes loss-of-function experiments in adult human cells. We therefore designed a gain-of-function approach, using transfection into primary fibroblast cells that do not normally express these genes. By using ectopic expression of ETCHbox genes in fibroblasts followed by high-throughput RNA-seq, we uncovered dramatic transcriptional changes driven by these genes. Most strikingly, many of the ETCHbox downstream genes that changed in expression (up or down) belong to a particular set of genes with a shared temporal profile in the human embryo, characterised by a sharp ‘pulse’ of high expression at the 8-cell to morula stages. This is precisely the stage of development when the ETCHbox genes themselves are expressed. This finding suggests that the ectopic expression experiment has most likely recapitulated some aspects of in vivo biological roles and allows us to gain insights into the functions of these newly evolved homeobox genes.

We extract two main biological conclusions from the up- and down-regulated gene sets. First, we find that *ARGFX* is acting antagonistically to *LEUTX* and *TPRX1* genes; together, these genes may shape the rapid on/rapid off temporal profile of target genes, peaking just before the first cell fate decisions. The deployment of antagonistic regulators to effect precise modulation is a common feature of biological systems. Second, we identify putative downstream effectors, which may include direct and indirect transcriptional targets involved in developmental processes in humans. These include the gene encoding histone H2 variant *HIST1H2BD* and the *RELB* gene encoding a transcription factor in the NFkB pathway. Other developmentally-relevant genes up-regulated by *ARGFX* and down-regulated by either *LEUTX* or *TPRX1* include the TGFβ-responsive *RHOB* gene and a gene encoding a signalling molecule *HBEGF*. Expression profiles of several of these genes differ between human, cow and mouse, suggesting some target genes may differ across mammals (data not shown). Nonetheless, even though the kinetics of early development and genome activation differ between mammalian species, we detect a strong signal of conservation of downstream activity in cow, with 58 % of comparable bovine genes (19/33) having similar expression profiles to human.

It is striking that the evolutionary origin of most, and possibly all, ETCHbox genes dates precisely to the stem lineage of eutherian mammals. This date is inferred from finding orthologues of *Argfx*, *Dprx*, *Leutx*, *Tprx1* and *Tprx2* in both the Atlantogenata and Boreoeutheria clades, and a *Pargfx* gene or pseudogene in Boreoeutheria (Euarchontoglires and Laurasiatheria). None are found in monotremes or marsupials. The origin of the genes represents a particularly clear example of ‘asymmetric’ evolution, whereby after tandem gene duplication, one gene diverges little in sequence and retains the original role, while daughter genes diverge in sequence and function [14]. In this case, the progenitor gene is *Crx*, which retained its retinal function in mammals and changed little in amino acid sequence from *Crx* of other vertebrates; the daughter genes, *Argfx*, *Dprx*, *Leutx*, *Tprx* and *Pargfx*, diverged greatly in sequence and were recruited for novel early embryonic roles. The *Crx* gene in non-mammalian species, notably *Xenopus* and dogfish, is expressed in the early embryo in addition to the developing eye [35–37]. It is possible, therefore, that early embryonic expression was shared by the *Crx* gene early in mammalian evolution when its tandem duplicates were formed, facilitating recruitment of the tandem duplicates to early embryonic expression. Asymmetric evolution is not seen in all cases of homeobox gene duplication, with many examples known of subtle evolutionary changes to daughter genes after a duplication events [3, 38, 39]. It will be interesting to clarify the situations under which symmetric versus asymmetric evolution of transcription factor genes is favoured.

It would be wrong, however, to characterise the origin of ETCHbox genes as a simple case of tandem duplication and divergence. Our comparative analyses of the genomic regions around these genes reveals a far more dynamic picture. Many mammals have lost genes, including the human lineage, which lost *Pargfx*. Elephant, guinea pig, horse, bat, cow and pig have duplicated ETCHbox genes further, with murid rodents having an extreme combination of gene loss and additional duplication. In addition, we detect evidence for ghost ETCHbox loci, inferred from the presence of characteristic CNEs without associated genes. Furthermore, in at least two cases, the parental *Crx* gene has continued to spawn duplicates, with recent pseudogenes found in tenrecs and murid rodents. Also nearby are other multigene families, including the extensive leukocyte receptor complex, a dynamic cluster of immunoglobulin-like receptor genes with copy number variation in humans [22], and large arrays of C2H2 zinc finger genes [40]. The different gene components between species may seem in conflict with proposed critical roles in early developmental events; this may be reconciled by the partial redundancy we detect between *LEUTX* and *TPRX1* in humans, and the observation that all placental mammals analysed retain at least one *Tprx* gene copy.

Taken together, these data paint a picture of a complex and unstable chromosomal region that has been expanding and contracting extensively since the origin of placental mammals, spawning and deleting genes in its wake. The mechanism is unknown, but may be related to the low density of recombination hotspots in this region (the long arm of human chromosome 19), which may facilitate unequal cross-over [10, 41], together with a high density of long interspersed nuclear elements that could promote tandem gene duplication [40, 42]. Human chromosome 19 also has an elevated GC content [40, 43]. Whatever the mechanisms, we propose that this chromosomal region has been a hotspot for tandem gene duplication and gene loss for over 70 million years. We liken this unusual genomic region to a site of tectonic activity, where geologically unstable regions spawn or swallow the earth’s crust. One important result of ‘genomic volcanism’ was the birth of the ETCHbox genes, which were recruited for novel roles in mammalian embryogenesis, facilitating the formation of sophisticated extra-embryonic membranes necessary for internal development in placental mammals.

## Conclusions

In this study, we explored the origin, evolutionary fate and cellular function of a set of divergent homeobox genes. We show that these genes (*Argfx*, *Dprx*, *Leutx*, *Pargfx* and *Tprx*) are related to each other and arose by tandem duplication of the *Crx* gene followed by asymmetric divergence. Comparative genomic analysis reveals that these genes arose in the evolution of eutherian mammals as part of dynamic expansion and contraction on an unstable chromosomal region, leading to different gene complements in diverse mammalian species. In humans and cows, the genes are expressed primarily at preimplantation stages. Using ectopic expression, we show that the human genes encode nuclear proteins that activate and repress the expression of many downstream genes, with partial redundancy between *TPRX1* and *LEUTX*, and antagonism with *ARGFX*. The downstream human genes include several expressed in a pulse at 8-cell to morula, consistent with a model in which *ARGFX*, *TPRX1* and *LEUTX* regulate gene expression changes that presage the distinction of extra-embryonic and embryonic cell types in the development of humans and possibly other placental mammals.

## Methods

### Comparative genomics

Genome assemblies were accessed through the NCBI, UCSC and Ensembl webpages; search for ETCHbox genes used a range of BLAST algorithms both genome-wide and within syntenic regions. ETCHbox gene annotations were manually curated using information from cross-species comparisons, retroposed copies and, for human, RNA-seq expression data. The first coding exons of ETCHbox genes were found to be extremely short; therefore, we only identified them in humans and in species with recent retrocopies. For phylogenetic analysis, we aligned homeodomain and Otx-specific domain amino acid sequences (Fig. 3), homeodomain amino acid sequences (Additional file 4: Figure S3), or *Leutx* amino sequences and *Tprx* nucleotide sequences (Fig. 2) using MAFFT [44] as implemented in Jalview [45]. We built Maximum Likelihood trees with MEGA5 [46], under complex models (WAG + I + Γ and GTR + I + Γ) and performed 1000 bootstrap replicates. *Crx* and *Tprx* CNEs were detected with VISTA [47] using human *CRX* as a reference sequence, AVID as the alignment program, and the following parameters: 75 bp window, 70 % identity across 70 bp for human duplicates; 65 % identity, 70 bp for turtle and coelacanth. phastCons [27] scores for each nucleotide position corresponding to coding regions of human *CRX* and ETCHbox genes (excluding the first coding exons, which could not be identified in most species as indicated above) were obtained from the human NCBI36/hg18 Placental Mammal phastCons conservation track in UCSC using the Table Browser.

### Expression profiles

Publicly available human RNA-seq datasets (Additional file 9: Table S2) were aligned to the human reference genome NCBI GRCh38.p2 using the STAR RNA-seq aligner [48] using the default settings with the addition of --outSAMstrandField intronMotif. Human gene models for *ARGFX*, *DPRX*, *LEUTX*, *TPRX1* and *TPRX2* were replaced with corrected models determined from transcript data (Additional file 2: Figure S2), before fragments per kilobase per million reads (FPKM) values were generated using Cufflinks. Publicly available bovine RNA-seq datasets (Additional file 9: Table S2) were aligned to Ensembl reference genome UMD3.1 using Tophat (default settings). Cow gene models for *Dprx*, *Tprx1* and *Tprx2* were added to the annotation and FPKM values generated using Cufflinks.

### Ectopic expression

Human *ARGFX* coding sequence was synthesized by GenScript USA, *DPRX* and *LEUTX* were cloned by ligating products of exon-specific genomic PCRs and *TPRX1* was generated by ligating a 5’ *TPRX2* region with 100 % identity to *TPRX1* with *TPRX1*-specific 3’-region amplified from human DNA. All coding sequences were cloned in-frame with a C-terminal V5 tag under control of a CMV promoter (pSF-CMV-Puro-COOH-V5, Oxford Genetics #OG3422). Primary human dermal fibroblasts were obtained from ScienCell (#2320); third passage cells grown in fibroblast medium (ScienCell #2301) were combined with endotoxin-free expression constructs in a 2 mm gap and electroporated using a NEPA21 Super Electroporator. One sample for each construct was processed for immunofluorescence after 48 h culture: cells were fixed (4 % formaldehyde, 10 min), Triton X-100 permeabilized (0.25 %, 10 min), BSA blocked (1 %, 30 min), reacted with mouse-anti-V5 antibody (ThermoFisher #37-7500; 1 h) and stained with secondary antibody (Alexa Fluor® 594, ThermoFisher; 1 h). Cells were co-stained with phalloidin (Alexa Fluor® 488, ThermoFisher) and DAPI (ThermoFisher) to visualise actin and DNA, and visualised using an inverted IX81 motorized microscope equipped with FV1000 Point scanning laser and Becker and Hickel FLIM system. Transfection efficiency was determined by counting V5-immunostained fibroblast nuclei as a percentage of DAPI-stained nuclei across three randomly selected fields of view. Full length protein translation was assessed by western blotting as follows. Total protein was extracted using RIPA buffer supplemented with cOmplete mini protease inhibitor (Roche #04693124001); 30 μg protein per sample. Western blots were probed with HRP-conjugated anti-V5 antibody (ThermoFisher #R961-25). For RNAseq, three biological replicates were processed for RNA extraction after 48 h using an RNeasy Mini Kit (Qiagen). A standard 1 μg of RNA was used in mRNAseq library preparation using the TruSeq RNA kit (Illumina) at the Oxford Genomics Centre in the Wellcome Trust Centre for Human Genetics, University of Oxford. The 15 libraries were multiplexed and split across two HiSeq4000 lanes in 75 bp paired-end mode, giving a total of 543 million paired-end reads (range 20.8 to 55.1 million per sample). Reads from each library were aligned to the human reference genome NCBI GRCh38.p2 using the STAR RNA-seq aligner [48]. Differential gene expression analyses were conducted using raw read counts in DESeq2 [49]. In addition, FPKM values were calculated for each gene model, as described above. Criteria used to delineate a confident set of differentially expressed genes in each condition, relative to control, were: (1) adjusted *P* value (Benjamini–Hochberg correction) less than 0.05, (2) expression level greater than 2 FPKM, and (3) fold-change greater than 1.25, equivalent to 2-fold change per cell with 25 % transfection efficiency. RNA-seq raw sequence files have been deposited in the NCBI Gene Expression Omnibus (www.ncbi.nlm.nih.gov/geo) under accession GSE80282. Additional file 10: Table S3 gives raw read counts per gene in each sample and the mean across replicates. Additional file 11: Table S4 gives FPKM counts per gene in each sample and the mean across replicates. Additional file 12: Table S5 gives differential expression statistics for all genes significant in at least one condition.

### Enrichment in embryonic temporal profiles

Gene expression data from seven human developmental time points (oocyte, zygote, 2-cell, 4-cell, 8-cell, morula, and blastocyst; Additional file 13: Table S6) were clustered using Mfuzz [50] including all genes with corrected reads per kilobase of interrogated region per total million mapped reads (cRPKM) > 1 and variance > 5. Applying a Pearson correlation of 0.95 yielded 106 distinct temporal profiles of expression. Of these, 28 profiles show an increase at genome activation (4-cell to 8-cell). To determine if any of the 106 profiles were enriched in genes activated or repressed in the ectopic expression experiment, we first removed genes from the ectopic datasets not represented in the embryonic profiles, and the transfected genes themselves, and tested for enrichment using χ^2^ test (*P* < 0.05 with false discovery adjustment). Gene expression data for profile 27 orthologues in cow across six developmental time points (oocyte GV (Germinal vesicle), oocyte MII (Metaphase II), 4-cell, 8-cell, 16-cell, and blastocyst; Additional file 13: Table S6) were clustered using Mfuzz [50], removing genes not expressed in cow early development, generating five unique profiles.

## Abbreviations

CNE, conserved non-coding element; ETCHbox, eutherian totipotent cell homeobox; FPKM, fragments per kilobase per million reads; hESC, human embryonic stem cell

## Additional files

Additional file 1: Figure S1. Synteny evidence for orthology of *Argfx*, *Dprx*, *Leutx*, *Pargfx* and *Tprx* loci between placental mammals. (A) Overview of synteny as deduced by identification of blocks of sequence similarity (grey lines) between chromosomes from four placental mammals and one marsupial. Although overall synteny is present between placental and marsupial mammals, the *Argfx*, *Dprx*, *Leutx*, *Pargfx* and *Tprx* loci (coloured boxes) are only present in placental mammals. (B–E) Detailed synteny analyses showing neighbouring loci (unfilled arrows) close to: (B) *Crx* (black) and *Tprx* (red) genes; (C) *Argfx* (purple); (D) *Dprx* (blue) and *Pargfx* (orange); and (E) *Leutx* (green) genes. The symbol Ψ indicates that loci are putative pseudogenes; arrowheads indicate presence of miRNA genes not their number or orientation. In panel B, dashed arrows on opossum chromosome 4 indicate gaps in the assembly that impeded identification of these loci in the corresponding regions; information from Tasmanian devil and wallaby genomes suggests that both loci are present in marsupials. In panel E, *Selv* genes, which are placental-specific paralogs of the *Crx* neighbour gene *Sepw1*, are depicted in grey; the number and orientation of *Lgals*/*Clc* and *Klk* genes is not shown. (PDF 257 kb) Additional file 2: Figure S2. Pargfx alignment, Otx-specific domain, conserved non-coding elements and full human coding sequences. (A) Homeodomain and C-terminal region of deduced Pargfx proteins of (from top) white rhinoceros, horse, dog and cat, pseudogene from ferret, and retroposed pseudogene from human. (B) Protein sequence alignment of the C-terminal part (downstream of the homeodomain) of different Otx-related proteins, including the placental-specific *Argfx*, *Leutx* and *Pargfx*. Shaded positions indicate > 65 % sequence identity. (C) Nucleotide alignments of *Crx* CNEs from several vertebrates plus the four to five paralogous copies of the same element from three placental mammals. Nucleotide shadowing intensity correlates with sequence conservation. Placental CNE copies are named as in Fig 3b. (D) Deduced coding regions of human *ARGFX*, *DPRX*, *LEUTX*, *TPRX1*, *TPRX2.* Lower case regions indicate repetitive DNA in coding sequence as inferred from UCSC repeat masker. Species abbreviations: Bflo, Florida amphioxus (*Branchiostoma floridae*); Btau, cow (*Bos taurus*); Lcha, coelacanth (*Latimeria chalumnae*); Cfam, dog (*Canis familiaris*); Chof, sloth, (*Choloepus hoffmanni*); Cpic, painted turtle (*Chrysemis picta*); Cpor, guinea pig (Cavia porcellus); Csim, white rhinoceros (*Ceratotherium simum*); Dnov, armadillo (*Dasypus novemcinctus*); Ecab, horse (*Equus caballus*); Fcat, cat (*Felis catus*); Hgla, naked mole-rat (*Heterocephalus glaber*); Itri, ground squirrel (*Ictodomys tridecemlineatus*); Lafr, elephant (*Loxodonta africana*); Hsap, Hsa, human (*Homo sapiens*); Mdom, opossum (*Monodelphis domestica*); Mput, ferret (*Mustela putorius*); Oana, platypus (*Ornithorhynchus anatinus*); Pcap, hyrax (*Procavia capensis*); Sara, shrew (*Sorex araneus*); Sscr, pig (*Sus scrofa*). (PDF 336 kb) Additional file 3: Table S1. ETCH box gene, pseudogene and retrocopy complements in placental mammal species. We classify a locus as a probable pseudogene if it contains mutations (frame shifts, stop codons and losses of splice sites) that disrupt translation initiation and/or presence of the homeodomain; we did not include C-terminal truncations but these are marked with an asterisk. (XLSX 12 kb) Additional file 4: Figure S3. Phylogenetic test of homology between *Crxos* and *Obox* genes with *Tprx* genes. Inclusion of sequences from naked mole-rat and guinea pig bridged the phylogenetic gap between murid rodents and other mammals, revealing a *Tprx1* gene clade (including the mouse *Crxos* gene) and a *Tprx2* gene clade (including mouse *Obox* loci). (PDF 199 kb) Additional file 5: Figure S4. Western blot of V5-tagged proteins. Total protein extracts from fibroblasts transfected with either empty, ARGFX-V5, DPRX-V5, LEUTX-V5, or TPRX1-V5 constructs were probed with an anti-V5 antibody. (PDF 907 kb) Additional file 6: Figure S5. Genes peaking in human 8-cell and morula regulated by ETCHbox expression. Overlap between Venn diagram showing names of genes up- or down-regulated by ectopic expression of *ARGFX*, *LEUTX* or *TPRX1* in fibroblasts and which are also present in temporal expression profile 27 of human developmentally-expressed genes. (PDF 26 kb) Additional file 7: Figure S6. Analysis of cow orthologues of human profile 27 genes. Nested circles show that of the 50 genes in human expression profile 27 and also affected by ETCHbox expression, 46 genes have orthologues in cow and of these 33 are expressed between oocyte and blastocyst in cow. Mfuzz clustering of the expression profiles of the 33 genes gave five profiles of which three are similar to human profile 27 with peak expression at 8-cell and/or 16-cell stages (clusters A, B, C). These three profiles contain the 19 bovine genes listed. GV, Germinal vesicle; MII, metaphase II oocyte; 4C, 4-cell; 8C, 8-cell; Bl, blastocyst. (PDF 346 kb) Additional file 8: Figure S7. Search for antagonistic effects between ARGFX, LEUTX and TPRX1. Venn diagrams examining possible antagonistic effects assessed by the identity of genes affected by ectopic expression. The top left panel (boxed) shows the strongest evidence for antagonistic effects, with 124 genes up-regulated by *ARGFX* ectopic expression but down-regulated by both *LEUTX* and *TPRX1*. (PDF 445 kb) Additional file 9: Table S2. Human and bovine adult and developmental data used for expression profiling. Sequence Read Archive database accessions for RNA-seq datasets used for expression profiling of genes in Fig. 4b and c. (XLSX 26 kb) Additional file 10: Table S3. Raw read counts after ectopic expression. Raw read counts from HiSeq4000 output mapped to human gene models excluding transfected genes; not normalised for sequencing depth or gene length. Counts for each replicate sample and means are provided. (XLSX 3221 kb) Additional file 11: Table S4. Fragments per kilobase per million reads (FPKM) values after ectopic expression. FPKM mapped for all human gene models excluding transfected genes. Counts for each replicate sample and means are provided. (XLSX 4778 kb) Additional file 12: Table S5. Genes affected by ectopic expression. List of genes with differential expression triggered by ectopic expression of ETCHbox genes as determined by DESeq2. (XLSX 401 kb) Additional file 13: Table S6. Human and cow RNAseq data used for temporal profiling. Sequence Read Archive (SRA) database accessions for human RNA-seq datasets used for identifying the 106 distinct temporal profiles of human genes expressed in the early embryo; SRA database accessions for bovine RNA-seq datasets used for identifying the five temporal expression profiles of bovine orthologues of human profile 27/ETCHbox-responsive genes. (XLSX 16 kb)
