# Supplementary figures and images for: Evolutionary origin and functional divergence of totipotent cell homeobox genes in eutherian mammals
Source: BMC Biol. 2016 Jun 13;14:45. doi: 10.1186/s12915-016-0267-0 (PMC4904359; doi:10.1186/s12915-016-0267-0)

A

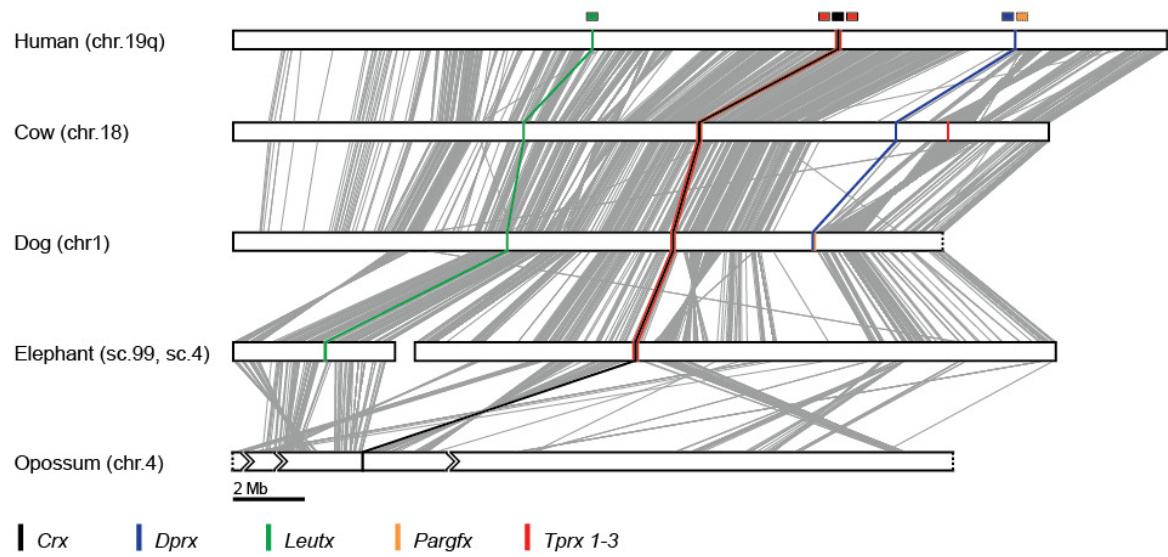

B

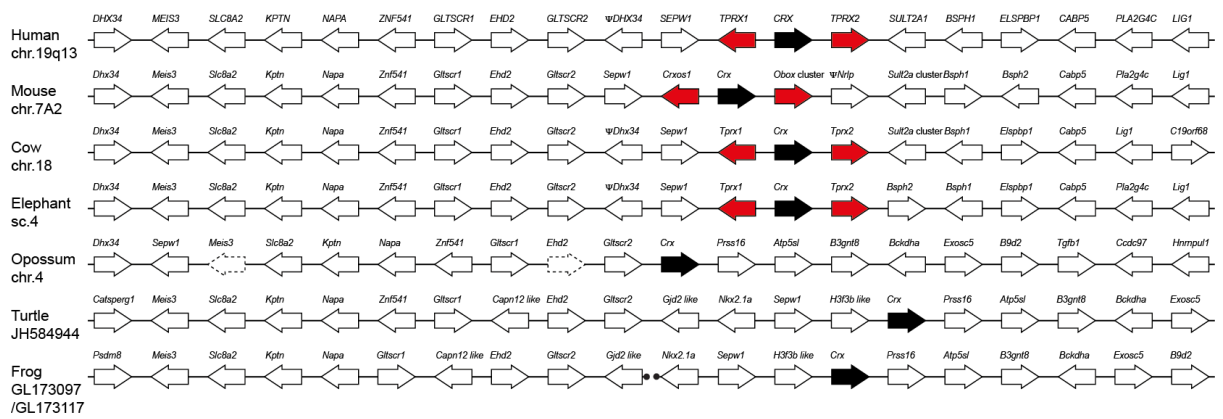

C

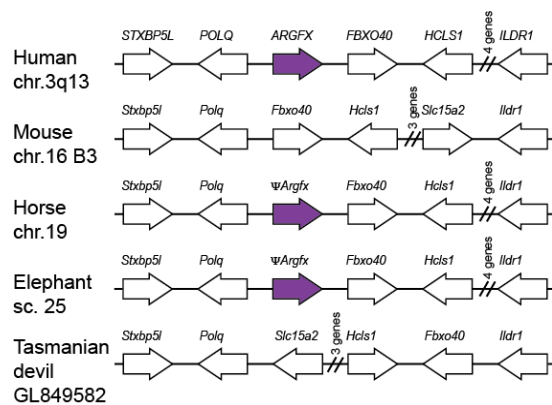

D

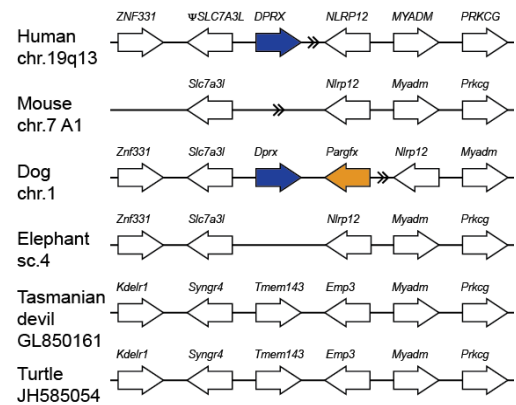

E

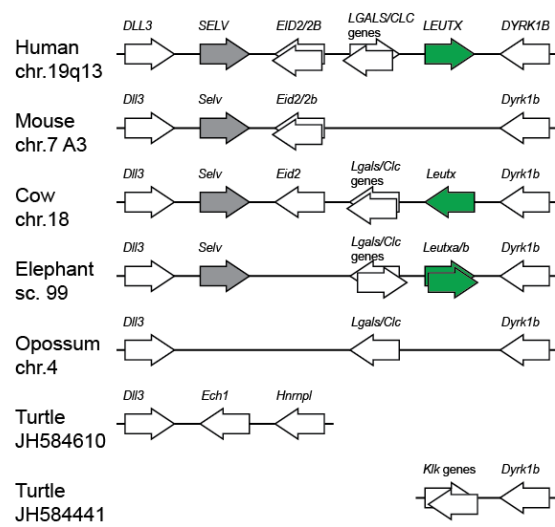

Figure S1

Supplement: Additional file 1: Figure S1. — Synteny evidence for orthology of Argfx, Dprx, Leutx, Pargfx and Tprx loci between placental mammals. (A) Overview of synteny as deduced by identification of blocks of sequence similarity (grey lines) between chromosomes from four placental mammals and one marsupial. Although overall synteny is present between placental and marsupial mammals, the Argfx, Dprx, Leutx, Pargfx and Tprx loci (coloured boxes) are only present in placental mammals. (B–E) Detailed synteny analyses showing neighbouring loci (unfilled arrows) close to: (B) Crx (black) and Tprx (red) genes; (C) Argfx (purple); (D) Dprx (blue) and Pargfx (orange); and (E) Leutx (green) genes. The symbol Ψ indicates that loci are putative pseudogenes; arrowheads indicate presence of miRNA genes not their number or orientation. In panel B, dashed arrows on opossum chromosome 4 indicate gaps in the assembly that impeded identification of these loci in the corresponding regions; information from Tasmanian devil and wallaby genomes suggests that both loci are present in marsupials. In panel E, Selv genes, which are placental-specific paralogs of the Crx neighbour gene Sepw1, are depicted in grey; the number and orientation of Lgals/Clc and Klk genes is not shown. (PDF 257 kb) [file 12915_2016_267_MOESM1_ESM.pdf]

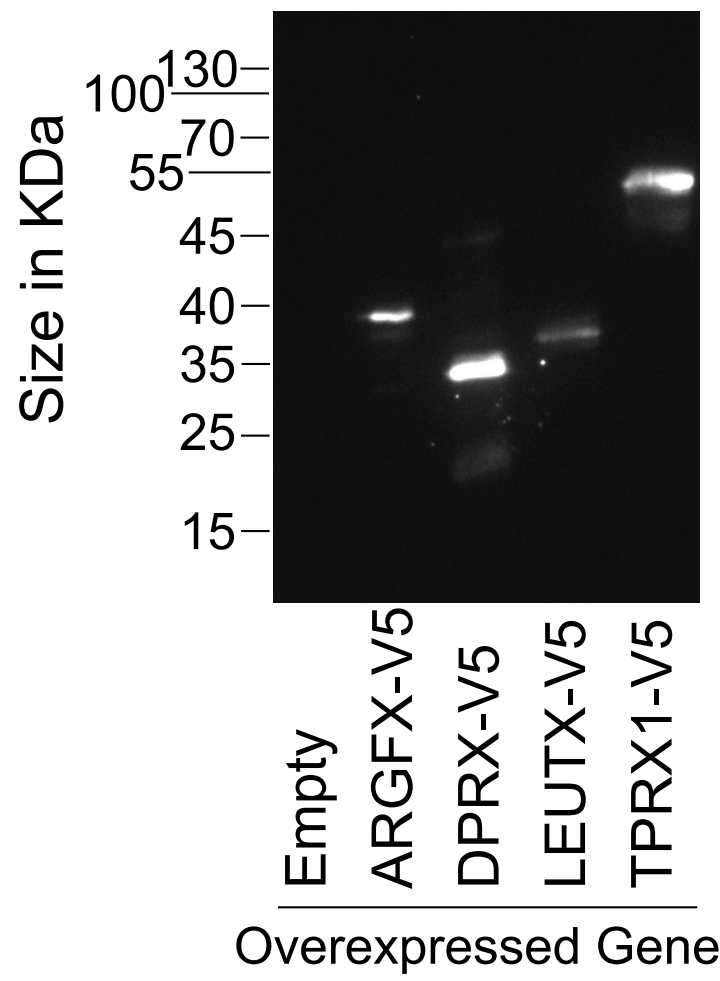

Supplement: Additional file 5: Figure S4. — Western blot of V5-tagged proteins. Total protein extracts from fibroblasts transfected with either empty, ARGFX-V5, DPRX-V5, LEUTX-V5, or TPRX1-V5 constructs were probed with an anti-V5 antibody. (PDF 907 kb) [file 12915_2016_267_MOESM5_ESM.pdf]

# CLUSTER 27 OVERLAP

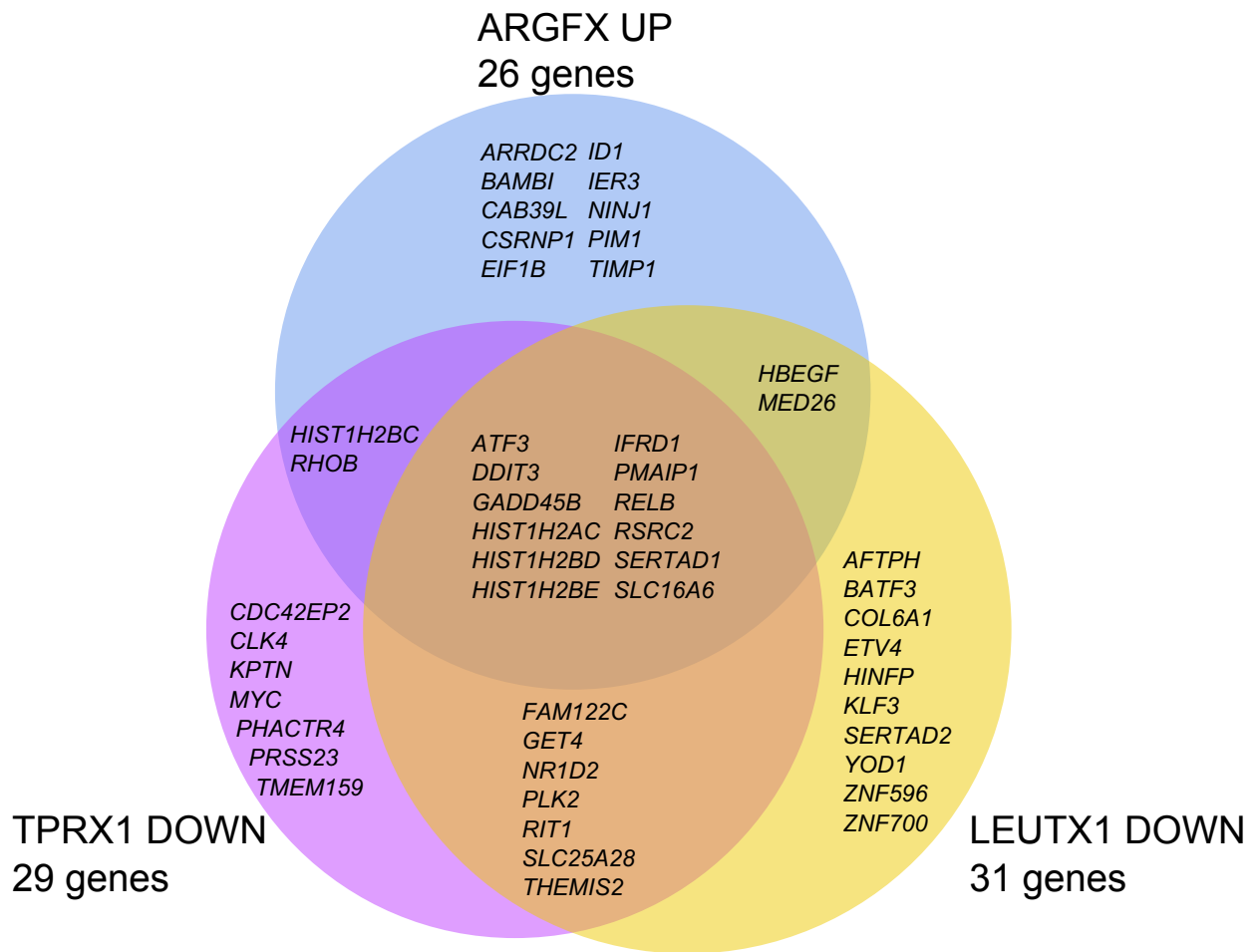

Supplement: Additional file 6: Figure S5. — Genes peaking in human 8-cell and morula regulated by ETCHbox expression. Overlap between Venn diagram showing names of genes up- or down-regulated by ectopic expression of ARGFX, LEUTX or TPRX1 in fibroblasts and which are also present in temporal expression profile 27 of human developmentally-expressed genes. (PDF 26 kb) [file 12915_2016_267_MOESM6_ESM.pdf]
