# Supplementary material for: Evolutionary origin and functional divergence of totipotent cell homeobox genes in eutherian mammals
Source: BMC Biol. 2016 Jun 13;14:45. doi: 10.1186/s12915-016-0267-0 (PMC4904359; doi:10.1186/s12915-016-0267-0)
Supplement: Additional file 2: Figure S2. — Pargfx alignment, Otx-specific domain, conserved non-coding elements and full human coding sequences. (A) Homeodomain and C-terminal region of deduced Pargfx proteins of (from top) white rhinoceros, horse, dog and cat, pseudogene from ferret, and retroposed pseudogene from human. (B) Protein sequence alignment of the C-terminal part (downstream of the homeodomain) of different Otx-related proteins, including the placental-specific Argfx, Leutx and Pargfx. Shaded positions indicate > 65 % sequence identity. (C) Nucleotide alignments of Crx CNEs from several vertebrates plus the four to five paralogous copies of the same element from three placental mammals. Nucleotide shadowing intensity correlates with sequence conservation. Placental CNE copies are named as in Fig 3b. (D) Deduced coding regions of human ARGFX, DPRX, LEUTX, TPRX1, TPRX2. Lower case regions indicate repetitive DNA in coding sequence as inferred from UCSC repeat masker. Species abbreviations: Bflo, Florida amphioxus (Branchiostoma floridae); Btau, cow (Bos taurus); Lcha, coelacanth (Latimeria chalumnae); Cfam, dog (Canis familiaris); Chof, sloth, (Choloepus hoffmanni); Cpic, painted turtle (Chrysemis picta); Cpor, guinea pig (Cavia porcellus); Csim, white rhinoceros (Ceratotherium simum); Dnov, armadillo (Dasypus novemcinctus); Ecab, horse (Equus caballus); Fcat, cat (Felis catus); Hgla, naked mole-rat (Heterocephalus glaber); Itri, ground squirrel (Ictodomys tridecemlineatus); Lafr, elephant (Loxodonta africana); Hsap, Hsa, human (Homo sapiens); Mdom, opossum (Monodelphis domestica); Mput, ferret (Mustela putorius); Oana, platypus (Ornithorhynchus anatinus); Pcap, hyrax (Procavia capensis); Sara, shrew (Sorex araneus); Sscr, pig (Sus scrofa). (PDF 336 kb) [file 12915_2016_267_MOESM2_ESM.pdf]

A

```
                      10        20        30        40        50        60        70        80
                      |         |         |         |         |         |         |         |
CsimPargfx   KPKRRSRERTSFTPDQKAELEALFSLTQFPDYATQELLASKIHLQFTVVQVWFKNRRVKWRKSEWKEQE-GLPTHAHPAKRRRTTAPRD
EcabPargfx   KPKRRSRERTSFTPDQKAELEALFSLTQFPDYVTQESLASKIHLQYSVVQVWFKNRRVKWRKSEWKVRE-GLPARACPAKRKTATAPRD
CfamPargfx   KPKKRSRERTSFTSYQKAELEALFNLTQFPDYVTQELLASKIHLQYTVVQVWFKNRRVKWRKSIQKEQEAGLPSLAPAAKRKRALEHRH
FcatPargfx   KPKRRSRERTSFTSHQKAELEALFSLTQFPDYVTQELLASKIHLQYTVVQVWFKNRRVKWRKSIQKDQEAGIPAPTPAAQRNRPRAPRR
MputPargfxps AKLKRSRERTSFTSHQKAELEALFSLTQFPDYVTQESLASKINLQYTVVQVWFKNRXVKWRKSVEKGQEVGLPRPTPAARRNTAPAPTH
HsaPARGFXr   HQLNXSXEGTSVTTDLKAELEALFRLT---DYFTQQLLASRICLQFK-VXVWFKNCQVK-----XKVRKXDFKLXAYPAKXKQAMAPQH

             90        100       110       120       130       140       150       160       170
             |         |         |         |         |         |         |         |         |
CsimPargfx   LANRGPVLPRFSDPQSTPLPDQSEDCVGNPDWTSPQRDSELDGLVATVPALSSTPYDIAQVIEAYGCFEEDENSNTFTCLYQYLSLSDT
EcabPargfx   LADGGALLPRVPDPQSTPLTGCSEDCFPNPDGTSPQCDSELDGLVATVPALSSTPYDIAQVMEAYGCFEEAESSSTFTCLYQYLSLSDS
CfamPargfx   LVNSRPILPRLSHP--------PEAGPGNPDGASQQCDPELDKLVATVPALSTAPYDISQVMEAYGCLEEEESSNTFTCLYQYLSPSDS
FcatPargfx   LSHRRPVLPRVSDG--------SGPGTGNPDGASPQGDPELDRLVATVPALSSAPYDISQVMEAYGCL--EESSNTFSCLYQYLSLSDG
MputPargfxps LAHSRLTLPKCPRP---------------PEAGVGDCDPELDRLVATVPALSSAPYDISQVMEAYGCLGEEESSNTFTCLYQYLSQTEC
HsaPARGFXr   A--GGPVLPRPLDPYSTHLPGPSGAHIVSLGWTSQMEESELKRLVASL-ALS-----LDQVIEAYRCFEKDASSNTFNCLCSRLLA---
```

B

```
BfloOtx      AGQAKPRPKKKS--ASPPPASTEEQAPTSESPSCSDS-SVSSTPVPVAVTIGNTNASPTAAV-----------------------------STSSIWSPASAPSVTSD--
HsapOTX1     GSGTKSRPAKKK--SSPVRESSGS--------ESSGQ-FTPPAVSSS--------ASSSSSASSSSANPAAAAAAGLGGNPVAAASSLSTPAASSIWSPASISPGSAPAS
HsapOTX2     GGQNKVRPAKKK--TSPAREVSSES-------GTSGQ-FTPPSSTSV--------PTIASSS-----------------------------APVSIWSPASISPLSDPLS
MdomCrx      GGTSKARPAKKK--CVSPPRGGAEPGPE--PPG-------PYSPAPA--------PSATPPS-----------------------------ASVSIWSP-GLSPLSDP--
BtauCrx      GAQAKARPAKRK--AGTSPRSSTDVCPD--PLGISDS-YSPPLPGPS--------GSPTTAV-----------------------------ATVSIWSPASESPLPEA--
LafrCrx      GGSSKARPAKRK--AGTSPRASTDTCPD--PLGISDS-YSPPLPGPS--------ASPTTAV-----------------------------ATVSIWSPASESPLPEA--
HsapCRX      GGQAKARPAKRK--AGTSPRPSTDVCPD--PLGISDS-YSPPLPGPS--------GSPTTAV-----------------------------ATVSIWSPASESPLPEA--
CfamPargfx   GLPSLAPAAKRK--RALEHRHLVNSRPI--LPRLSHP-PEAGPG-----------------------------------------------------NPDGASQQCDP--
FcatPargfx   GIPAPTPAAQRN--RPRAPRRLSHRRPV--LPRVSDG-SGPGTG-----------------------------------------------------NPDGASPQGDP--
EcabPargfx   GLPARACPAKRK--TATAPRDLADGGAL--LPRVPDPQSTPLTGC----------------------------------------------SEDCFPNPDGTSPQCDS--
BtauArgfx    KPPSQVLPA-KD--V---PTVSTSPHSF--LLAISDS-YNSLSPQPL--------DTFPWAG-----------------------------DSMIMEIPTSDVQTQDP--
SscrArgfx    KPPNQTLLA-KK--T---PTASTSPHSF--FPEVSDS-SIFLSTRPL--------GPFQCAG-----------------------------NSTITESPTSDVQMQDP--
EcabArgfx    KQQNQILPA-KT--VPNSPTASSSPCSFV-XPAVSDF-CSSFXTSAL--------RPFPWAW-----------------------------DSIITESPTRDVQMQDL--
SaraArgfx    KMSKKKLLN-NK--LPNLPQQPTNAHTS--YPAVSDF-CSSSHTHPL--------GPSNCTQ-----------------------------DSIMTLSLPSDIQMPDT--
HsapARGFX    KQRNQILPSKKN--VPTSPRTSPSPYAF--SPVISDF-YSSLPSQPL--------DPSNWAW-----------------------------NSTFTESSTSDFQMQDT--
HsapLEUTX    GPANQTTSVKKE--ETPSAITTANIRPV--SPGISDA-NDHDLREPS--------GIKNPGG-----------------------------ASASARVSSWDSQSYDI--
FcatLeutx    KSPNQTISIKEE--QPPLPVTSANTHPG--SPSILDA-CDHELPQSP--------DSVQSAG-----------------------------AGPSKWNSSWDSPPYDL--
EcabLeutx    GGPNQTTSAKEE--EAPLPVTSAATHLT--SPAVSDD-CGHEPPKPS--------GIEQPGG-----------------------------AGASVWN-SWDPQLQNL--
LafrLeutxA   EEPNHVAPVKKE--EMPLPITDANPGPV--SARSLDA-SEPDPPEPP--------GAGQPGG-----------------------------AGACVWNSSWDSHTHDA--
PcapLeutx1   EDLGQVVPVEEEEKETPLLTTDANALP---VGTRSYA-CKPCPPQQH--------ESGKPAG-----------------------------AGACVWNSSRDSETHDV--
ChofLeutx    EAPNQTVSVEKE--ETPLATTASRSSL---SPGISDA-RGPVPSEYP--------GTEQLNG-----------------------------AGASACISSWDSQPYDR--
DnovLeutx    EAPTKTVSVKEE--ETPLAITDAQSPP---SPGISDP-RNPETPEGS--------GSEHPKG-----------------------------AGASAENSSWNSRPCDL--

BfloOtx      -LGSCTSTVTSAPSCMQRS------------SYNHAYNHG--GYTAHAQTYSPAPYPPTTYSTSYFGGMADCSSYLSPM
HsapOTX1     VSVPEPLAAPSNTSCMQRS--VAAGAATAAASYPMSYGQG----GSYGQGYP------TP-SSSYFGGV-DCSSYLAPM
HsapOTX2     ----------TSSSCMQR-------------SYPMTYTQA----SGYSQGYA------G--STSYFGGM-DCGSYLTPM
MdomCrx      --LAAMGASGSSAVCTQRA---PGAPVAASSSYALTYAPAPAAFSASSSPYG------S--SSSFFGGL-DCSPYLSPM
BtauCrx      ----------------QRAGLVASGPSLTSAPYAMTYAPA-SAFCSSPSAYG------S--PSSYFSGL---DPYLSPM
LafrCrx      ----------------QRAGLVASGPALTSAPYAMTYAPA-SAFCSSPSAYG------S--PSSYFSGL---DPYLSPM
HsapCRX      ----------------QRAGLVASGPSLTSAPYAMTYAPA-SAFCSSPSAYG------S--PSSYFSGL---DPYLSPM
CfamPargfx   ----------------ELDKLVATVPALSTAPYDISQVME--AYGCLEEEE----------SSNTFTCL---YQYLSPS
FcatPargfx   ----------------ELDRLVATVPALSSAPYDISQVME--AYGCLEE------------SSNTFSCL---YQYLSLS
EcabPargfx   ----------------ELDGLVATVPALSSTPYDIAQVME--AYGCFEEAE----------SSSTFTCL---YQYLSLS
BtauArgfx    ----------------QLERLVASVPALYSDAFDITQIME--LYSVPDEDD---------ITNSSFYSL---YQYLSPT
SscrArgfx    ----------------QLERLVASVPALYSDAYDIDQIMK--LYSFPDDNE---------LDSSSFHSL---YQYLSPI
EcabArgfx    ----------------QLERLVASVPALYSDAYDIAQIMK--LYSFPDEDE---------ISSSSFQYL---YWYLSPT
SaraArgfx    ----------------QLESLEATVPALFPDSYDITQIME--LYSFPDE-E---------LTSSSFECL---YQYLSPQ
HsapARGFX    ----------------QWERLVASVPALYSDAYDIFQIIE--LYNLPDENE---------ISSSSFHCL---YQYLSPT
HsapLEUTX    ----------------EQICLGASNPPWASTLFEIDEFVK--IYDLPGEDD----------TSS----L---NQYLFPV
FcatLeutx    ----------------QQICLGDPDPPWASIPYDIDQLIQ--LYALPGDDD-----------------L---DQYLSPV
EcabLeutx    ----------------QQICLGVSDPPWASVPTDMDEFIQ--LYALPGDDD----------PSS----L---DEYLFPK
LafrLeutxA   ----------------QEINLDILDPPWATIPCDIDEFVE--LYALPGDDD----------PSS----L---DQYLLPR
PcapLeutx1   ----------------LEINLDVLSPPWAAAPCDIEEFVE--LYALPGDDD----------PSS----L---DQYLSPK
ChofLeutx    ----------------QEIFLGSSYPPWASILCDIDQFVE--LYALPGEDD----------PSN----F---DQYLFPK
DnovLeutx    ----------------QDKC-----PPWASILCDVERFVE--LYDLPGEVD----------PSE----L---DQYLFPE
```

C

```
LchaCNE          TTTTTACTTATCTAATTTAGTAAATGTTAAAATCCTCTTAAGGCCGC---TGCTCCCCTCAGCAGATTGGGTAACTGAATTAGTGAGGATTACAGTC
CpicCNE          TTTTTACTGATCTAATTTAGCAAATGTCCCAATCGGCTTAGGGCCG----CTGCCCCCTGCGCAGCTG-GGTAACTGAATTAGTGGGGATTAGGGAG
OanaCNE          TTTTTACTGATCTAATTTCACAAATGCTCCAATCCTCTTAGCCCAG----ACCTGGCCTCAGCACATT-GGTAAGTGAATTAGAGAGGATTGGGGGG
HsapCNE CRX      CCTGACCAGGACTAATTTAGCAAATGCTCCAATCCTCTTATGGCAACA--GCCCAGCCTCAGCAGAAT-GGCAGGGGAATTAGCGAGGATTAGGTTC
CfamCNE Crx      CCTGAACAGGACTAATTTAGCAAATGCCCCAATCTTCTTATGATG-----GTCCAGCCTCAGCAGAAT-GGCC-GGGAATTAGCAGGGATTAGGTTC
DnovCNE Crx      CCTGACCAGGCCTAATTTAGCAAATGCTCCGATCCGCTTA-GCCG-----GTGCAGCCTCAGCAGAAG-GGCG--GGGATTAGCGGGGCTTAGGGAC
HsapCNEa TPRX1   CCTGTCCCAGTCTCATTTAGCAAATGCTTCAATCCTCTTAAGTCA-----GGCCAGCCTCAGCAAAAC-ATAGGGAGAATTAGCGCGGATTTGGGGG
CfamCNEa Tprx1   CCTGTCCTGTTCCTACTTAGCAAATGCTTCAATCCTCTTAGGCCA-----GGCCAGCCTCAGCAAAAT-ATAGGGAGAATTAGCAGGGATTTGGAGG
DnovCNEa Tprx1   CCAGTTCTGCTCTCATTTAGCAAATGCTCCAATCCTCTTAGGCCG-----GG-CAGCCTCAGCAAAAC-TTAGAGAGCATTAGGGGGGATTTGTGGC
HsapCNEb TPRX2   CCTGTCCTGGTCCCGTTTAGCAAATGCTCTAATCCTCTTAGGCCA-----GGCCAGCCTCAGCAAAGC-AGAGAGAGAATTAGATCAGATGTTGGGG
CfamCNEb Tprx2   CCTGTCCTGGTCCCATTTACCAAATGCTCCAATCCTCTTAAGCCA-----GACCAGCCTCGGCAGAGC---AGAGAGAATTAGATCAGATTTGGGGG
DnovCNEb Tprx2   CCTGACGGGGTCTTATTTAGCAAATGCTCTAATCCTCTTAGGCCA-----GGCCAGCCTTGCAATAGC-AGAGAGAGACTTAGATGGTGTTTGGGGG
LafrCNEb Tprx2   CCTGTCCTGGACCCATTTAGCAAATGCTCTAATCCTCTTAGGCCA-----GGCCAGCCTCAGCAGAGC-AGAGAGAGAATAAGATAAGATTTGGGAG
HsapCNEc         CCTGTCCTGGTCCCATGTAGCAAGTGTTCCAGTCCTCTTAAGCCAG----GGCCAGTCTCAGCAGAGC-AGAGAGAGAATTAGATCCGATTTGGAGG
CfamCNEc         CCTGCCCTGGTCCCATTTAGCAAATGCTCCAATTTTTCAGGCCA-----GGCCAGCCTCAGCAGAAT-AGAGAGAAAATTAGATCAGATTGAGGGA
HsapCNEd         CCTGTCCTAGTCCCATTTAGCAAATGTTCCAATCCTCTTAGGCCAGGCCAGGCCAGCCTCAGCAGAGC-------AGAATTAGATCCGATTTGGGGG
CfamCNEd         CCTGTCCTAGTCCCATTTAGCAAATGCTCCAATCCTCTTAGGCTG-----GGCCCGCCTCAGCAGAGC-AGAGAGAGAATTAGATCAGATTTGGGGG
DnovCNEd         TCTGCCCTGAATTCATTTAGCAAATGCTCTAATCCTCTTAGGCC----------AGCCTCGGCAGAGC-AGAGAGAGAATTAGATGACATTTGGGGT
```

D

Human ARGFX coding sequence

Exon 1 (excluding 5′ untranslated region)
ATGAGGAACAGAATGGCCCCAGAGAATCCCCAGCCAGACCCTTTCATCAATAGGAATTATTCCAACATGAAGGTGATACCACCACA
GGATCCAGCTAGTCCCA

Exon 2 (lower case is part of Alu element)
gtttcactctgttatccaagctggagtgcagtggcacggtctcggcttactgcagcctcaacctcccaggttcaactgatcctccc
acctcagcctcccgagtagctgcgactacag

Exon 3
CAATACGGAGAAGGCATAAAGAACGTACTTCTTTCACCCACCAACAGTATGAGGAGCTAGAAGCTCTGTTTAGCCAGACCATGTTC
CCAGATAGAAATCTTCAGGAGAAACTAGCTTTGAGACTCGACCTACCGGAGTCAACAGTAAAG

Exon 4 (excluding 3′ untranslated region)
GTTTGGTTCAGGAACCGGCGATTCAAATTGAAGAAGCAGCAGCAGCAGCAATCAGCAAAGCAACGAAACCAGATCCTTCCATCCAA
GAAGAATGTGCCCACCTCCCCCAGAACATCCCCCAGTCCTTATGCTTTTTCTCCTGTGATTTCAGATTTCTACAGCTCCCTTCCAT
CTCAGCCCTTAGACCCTTCCAATTGGGCATGGAACTCTACCTTCACTGAGAGTTCTACCAGTGACTTCCAAATGCAAGATACTCAG
TGGGAGAGGCTGGTGGCCTCGGTTCCTGCTTTGTACTCTGATGCCTATGACATATTCCAAATCATAGAACTGTACAATCTTCCTGA
TGAGAATGAGATATCCAGCTCTTCTTTCCACTGTCTGTATCAGTATCTCTCACCCACAAAGTACCAGGTAGGAGGACAGGGTTCCT
CTCTCAGCATCTTTGCTGGTCCAGCTGTAGGCCTATCTCCTGCACAAACCTGGCCCAATATGACAAGCCAAGCCTTTGAAGCCTAC
AGTCTAACAGATAGCCTGGAATTCCAGAAAACCTCCAATATGGTAGACTTGGGATTTCTCTGA

Human DPRX coding sequence

Exon 1 (excluding 5′ untranslated region)
ATGCCAGGCTCAGAGGATCTTCGTAAAG

Exon 2
GCAAGGACCAGATGCATTCACACAGGAAACGAACCATGTTCACTAAGAAGCAACTGGAAGATCTGAACATCTTGTTCAATGAGAAC
CCATACCCAAACCCCAGCCTTCAGAAAGAAATGGCCTCGAAAATAGACATACACCCAACAGTACTGCAG

Exon 3 (excluding 3′ untranslated region)

GTCTGGTTCAAGAATCACAGAGCAAAACTCAAGAAAGCGAAATGCAAGCATATTCATCAAAAACAAGAAACTCCACAACCGCCAAT
ACCAGAGGGTGGGGTCTCCACCAGTGTCGGCCTGAGAAATGCAGACACACTACCCAGATTGCCCAACGCTGCTCACCCGATCGGCC
TGGTGTACACGGGTCATCGAGTCCCCTCATTCCAGCTCATCCTGTACCCCAACCTCAAGGTCCCTGCAAATGACTTCATTGGCCAC
AGAATAGTCCATTTTGGCTGCTGCCGAGATCCTAATATATACTGCCTCTACCCCATTTTGGAATCCCAAGTTTGCGCTCCAAGCTT
CCATTCTGGCTCTCCTGCCTGTTCATCTAACCAAAGTCGAGAGAGATGA

Human LEUTX coding sequence

Exon 1 (excluding 5′ untranslated region)
ATGTTTG

Exon 2
AAGGGCCAAGGCGTTATCGTCGGCCACGCACAAGATTTCTCTCCAAACAACTCACAGCATTGAGAGAATTGCTTGAAAAGACCATG
CACCCAAGTTTGGCTACAATGGGGAAACTGGCTTCAAAGCTACAACTTGATCTATCCGTAGTAAAG

Exon 3 (excluding 3′ untranslated region)
ATCTGGTTCAAGAACCAGCGTGCCAAATGGAAGAGGCAGCAGCGGCAGCAAATGCAGACACGGCCATCACTAGGGCCAGCAAACCA
GACAACTTCAGTGAAGAAGGAGGAGACTCCCTCAGCCATAACTACTGCAAACATTCGTCCAGTAAGTCCTGGAATCTCTGATGCAA
ATGACCATGATCTACGTGAGCCTTCTGGTATCAAGAATCCTGGAGGAGCCAGCGCCTCTGCGAGGGTTTCATCCTGGGATTCTCAG
TCATATGACATTGAACAGATATGTCTGGGGGCTTCAAATCCTCCTTGGGCCTCCACTCTCTTTGAAATAGATGAATTTGTAAAGAT
CTATGACTTGCCAGGGGAAGATGACACCAGCAGCCTAAATCAATATCTTTTTCCAGTATGCCTTGAGTATGACCAGCTCCAATCTT
CAGTGTAA

Human TPRX1 coding sequence

Exon 1 (excluding 5′ untranslated region)
ATGCAAGACCCTGGTCATCTCCAAG

Exon 2
GCCCTCCCCTGGCCCTGGACCCTCCAAGGAGACAGCGGCAGGAGCGCACGGTCTACACTGAAAGCCAGCAGAAAGTGCTAGAATTT
TACTTTCAGAAGGACCAGTACCCGAACTACGACCAGCGACTGAATCTGGCGGAGATGCTCAGCCTCAGGGAGCAACAGCTGCAG

Exon 3 (excluding 3′ untranslated region)
GTGTGGTTCAAGAATCGCCGCGCCAAACTAGCTCGGGAGCGGCGGCTCCAGCAGCAGCCCCAGCGCGTCCCTGGGCAGAGAGGCCG
AGGAGCCCGCGCTGCGCCCCTAGTCCCTGCAGCCTCTGCCTCCGCACCTCAGCGGGGCCCCTCGGGAATCCTTCCAGCGGCGGAAC
CCACGATCTGCAGCCTCCACCAGGCCTGGGGTGGCCCTGGGTGCAGAGCCCAGAAGGGCATCCCAGCTGCCCTGAGTCCAGGCCCT
GGCCCGATccctgccccaatcccaggcccagcccagatcccaggcccactccctggctcaattccaggcccaattccaggcccagc
tcagatcccaagcccgatcccagccccaatcccaggcccaatttcaggcccagtccagatcccaggcccattccgtggcccaatcc
caggcccaatttcaggcccagccccgatcccaggcccaatctcaggcccattctcaggcccaaacccaggcccgatcccaggccca
aacccaggcccgatcccaggcccaatctcaggcccgatcccaggcccaatctcagtcccgatcccaggcccgatcccaggcccaat
ctcaggcccaatctcaggcccaaacccaggcccgatcccaggcccaatcccaggcccaatctcaggcccgaacccaggcccgatcc
caggcccaatctcaggcccgaacccaggcctgatcccaggcccaatcccaggcccaatctcaggcccaggcccaATTATAGGCCCG
ATTCCCAGCCCAGCCCAGATCCCAGGCCCAGGCAGACTCCAAGGCCCAGGTCCCATCTTAAGTCCTGGCCGGATGCGAAGCCCTGG
CTCACTTCCAGGCCTAGCCCCGATTTTaggcccaggctcaggcccaggctcaggctcagtcccagctccaatcccaggcccaggat
cactcccagccccagccccCTTATGGCCTCAGAGCCCCGATGCCTCCGACTTCTTGCCAGACACCCAGTTATTCCCTCACTTCACA
GAGCTGCTCCTACCCCTAGACCCCTTGGAGGGATCCTCAGTCTCCACCATGACCTCTCAGTACCAAGAAGGGGATGACTCTATGGG
CAAAAAACACTCAGGGTCTCAGCCCCAAGAGGAGGGTGGCTCTGTGAATGAAAATCACTCAGGCCCCAGGTTATTACTGGATTTAT
AG

Human TPRX2 coding sequence

Exon 1 (excluding 5′ untranslated region)
ATGCAAGACCCTGGTCATCTCCAAG

Exon 2
GCCCTCCCCTGGCCCTGGACCCTCCAAGGAGACAGCGGCAGGAGCGCACGGTCTACACTGAAAGCCAGCAGAAAGTGCTAGAATTT
TACTTTCAGAAGGACCAGTACCCGAACTACGACCAGCGACTGAATCTGGCGGAGATGCTCAGCCTCAGGGAGCAACAGCTGCAG

Exon 3 (excluding 3′ untranslated region)
GTGTGGTTCAAGAATCGCCGCGCCAAACTAGCTCGGGAGCGGCGGCTCCAGCAGCAGCCCCAGCGCGTCCCTGGGCAGAGAGGCCG
AGGAGCCCGCGCTGCGCCCCTAGTCCCTGTAGCCGCTGCCTCCTTCCCTGGGGGTCCTGAGTTCCCGCAGGGCAGGGGTTCCTGGA
TCTCCCCTCAGCCGGGCCCCTGGGGAGTCCTCCCAGCAGCAGAACCGAAGATCTACAGCCTCCCCCGGACCTGGGGTGGCCCTGAG
TGTGGAACACAGGAGGGCCTCAAGGCTGTcccggctccaggccctggcccaatcccagcccctatcccaggcccagcccagatccc
aggcccagtcccaggcccagccccgaatttaggcccaatgtcaggcccactgtcagtctcgatccccggtccaataccagcccccca
tctcttgcccaggcccaatcccagacccagtcctaggccgaaccctgatgccaggcccaggatcactcccaaccccagccccagGC
GCCTTGTGGCCTCAGAGCCCCTATGCCTCCAACTTGTCGCCAGACACCCAGTTATACCCTGACTTCACCAAGCTGCTCCCGCTCCT
AGACCGGTTCGAGGAATCCTCACTCTCCACCACGACGTCTCAGTACAAAGAGGAGGATGGCTTCGTGGACAAAAATCACTCAGTCC
CCAGGTCATTACTGGATTTATAG
