# Supplementary material for: Evolutionary origin and functional divergence of totipotent cell homeobox genes in eutherian mammals
Source: BMC Biol. 2016 Jun 13;14:45. doi: 10.1186/s12915-016-0267-0 (PMC4904359; doi:10.1186/s12915-016-0267-0)
Supplement: Additional file 4: Figure S3. — Phylogenetic test of homology between Crxos and Obox genes with Tprx genes. Inclusion of sequences from naked mole-rat and guinea pig bridged the phylogenetic gap between murid rodents and other mammals, revealing a Tprx1 gene clade (including the mouse Crxos gene) and a Tprx2 gene clade (including mouse Obox loci). (PDF 199 kb) [file 12915_2016_267_MOESM4_ESM.pdf]

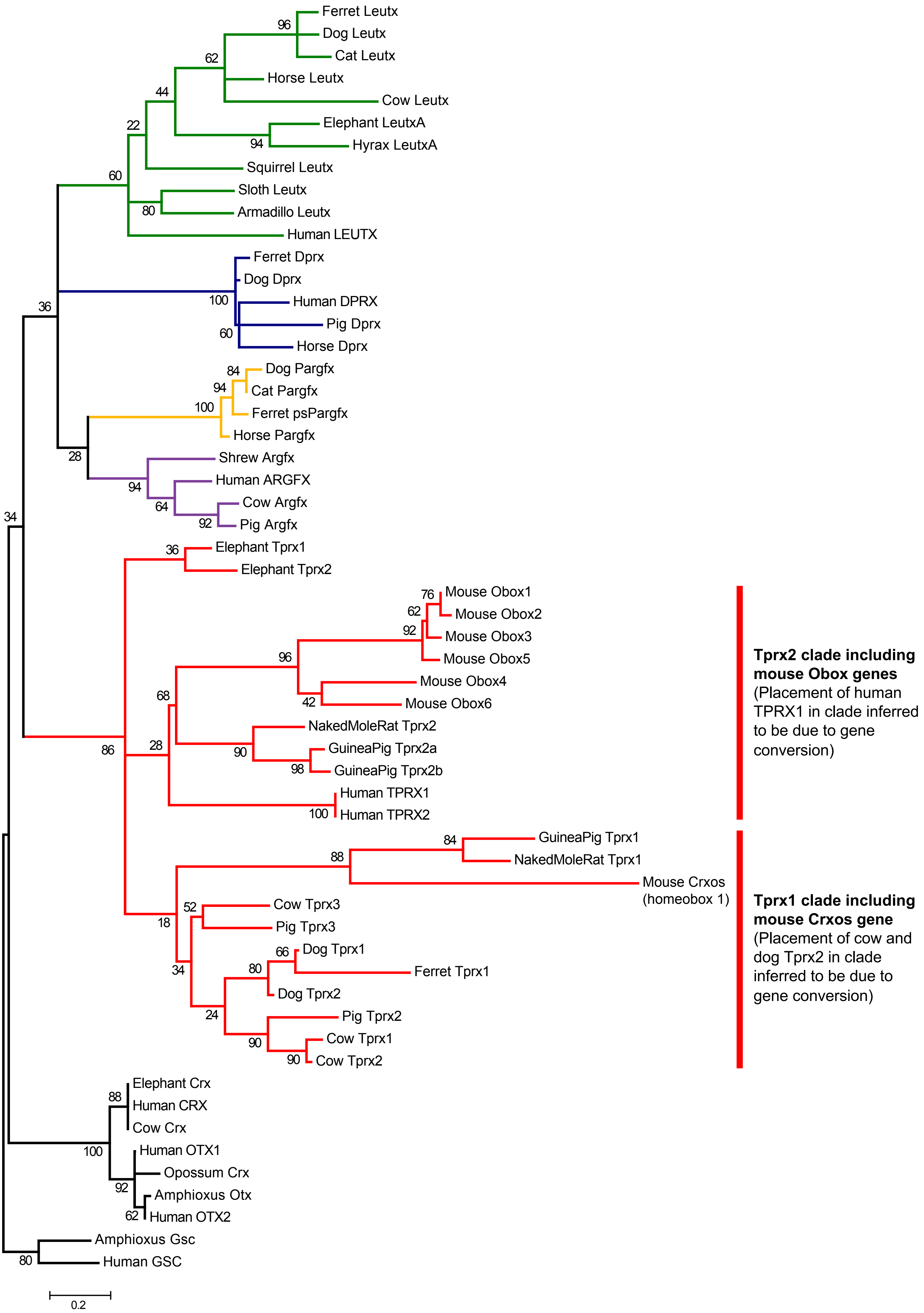
Ferret Leutx
Dog Leutx
Cat Leutx
Horse Leutx
Cow Leutx
Elephant LeutxA
Hyrax LeutxA
Squirrel Leutx
Sloth Leutx
Armadillo Leutx
Human LEUTX
Ferret Dprx
Dog Dprx
Human DPRX
Pig Dprx
Horse Dprx
Dog Pargfx
Cat Pargfx
Ferret psPargfx
Horse Pargfx
Shrew Argfx
Human ARGFX
Cow Argfx
Pig Argfx
Elephant Tprx1
Elephant Tprx2
Mouse Obox1
Mouse Obox2
Mouse Obox3
Mouse Obox5
Mouse Obox4
Mouse Obox6
NakedMoleRat Tprx2
GuineaPig Tprx2a
GuineaPig Tprx2b
Human TPRX1
Human TPRX2
GuineaPig Tprx1
NakedMoleRat Tprx1
Mouse Crxos
(homeobox 1)
Cow Tprx3
Pig Tprx3
Dog Tprx1
Ferret Tprx1
Dog Tprx2
Pig Tprx2
Cow Tprx1
Cow Tprx2
Elephant Crx
Human CRX
Cow Crx
Human OTX1
Opossum Crx
Amphioxus Otx
Human OTX2
Amphioxus Gsc
Human GSC
0.2
Tprx2 clade including mouse Obox genes
(Placement of human TPRX1 in clade inferred to be due to gene conversion)
Tprx1 clade including mouse Crxos gene
(Placement of cow and dog Tprx2 in clade inferred to be due to gene conversion)
