# Supplementary material for: Evolutionary origin and functional divergence of totipotent cell homeobox genes in eutherian mammals
Source: BMC Biol. 2016 Jun 13;14:45. doi: 10.1186/s12915-016-0267-0 (PMC4904359; doi:10.1186/s12915-016-0267-0)
Supplement: Additional file 7: Figure S6. — Analysis of cow orthologues of human profile 27 genes. Nested circles show that of the 50 genes in human expression profile 27 and also affected by ETCHbox expression, 46 genes have orthologues in cow and of these 33 are expressed between oocyte and blastocyst in cow. Mfuzz clustering of the expression profiles of the 33 genes gave five profiles of which three are similar to human profile 27 with peak expression at 8-cell and/or 16-cell stages (clusters A, B, C). These three profiles contain the 19 bovine genes listed. GV, Germinal vesicle; MII, metaphase II oocyte; 4C, 4-cell; 8C, 8-cell; Bl, blastocyst. (PDF 346 kb) [file 12915_2016_267_MOESM7_ESM.pdf]

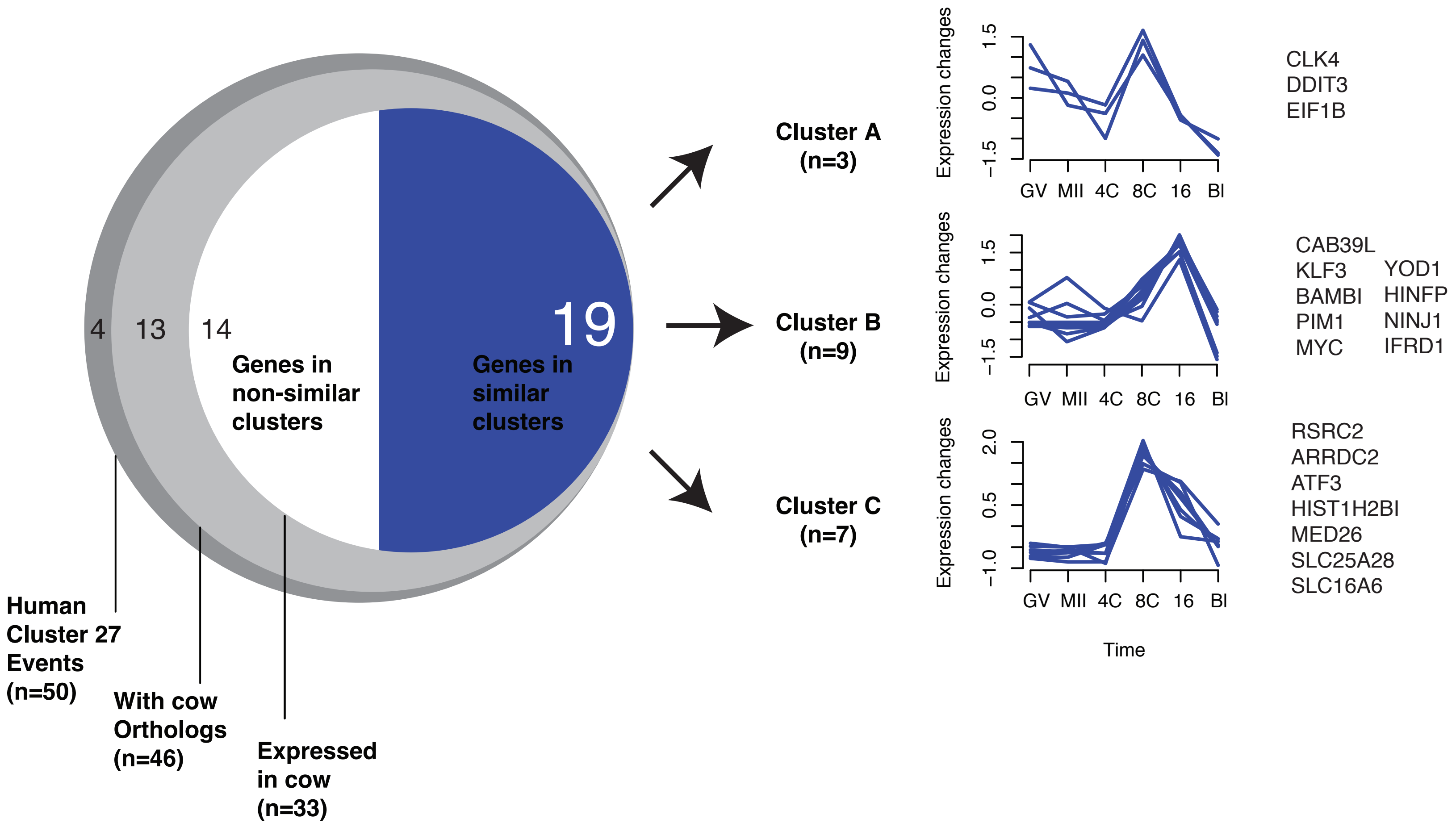

4
13
14
19
Genes in non-similar clusters
Genes in similar clusters
Human Cluster 27 Events (n=50)
With cow Orthologs (n=46)
Expressed in cow (n=33)
Cluster A (n=3)
Cluster B (n=9)
Cluster C (n=7)
Expression changes
GV MII 4C 8C 16 Bl
Time
CLK4
DDIT3
EIF1B
CAB39L
KLF3
BAMBI
PIM1
MYC
YOD1
HINFP
NINJ1
IFRD1
RSRC2
ARRDC2
ATF3
HIST1H2BI
MED26
SLC25A28
SLC16A6
