# Supplementary material for: Evolutionary origin and functional divergence of totipotent cell homeobox genes in eutherian mammals
Source: BMC Biol. 2016 Jun 13;14:45. doi: 10.1186/s12915-016-0267-0 (PMC4904359; doi:10.1186/s12915-016-0267-0)
Supplement: Additional file 8: Figure S7. — Search for antagonistic effects between ARGFX, LEUTX and TPRX1. Venn diagrams examining possible antagonistic effects assessed by the identity of genes affected by ectopic expression. The top left panel (boxed) shows the strongest evidence for antagonistic effects, with 124 genes up-regulated by ARGFX ectopic expression but down-regulated by both LEUTX and TPRX1. (PDF 445 kb) [file 12915_2016_267_MOESM8_ESM.pdf]

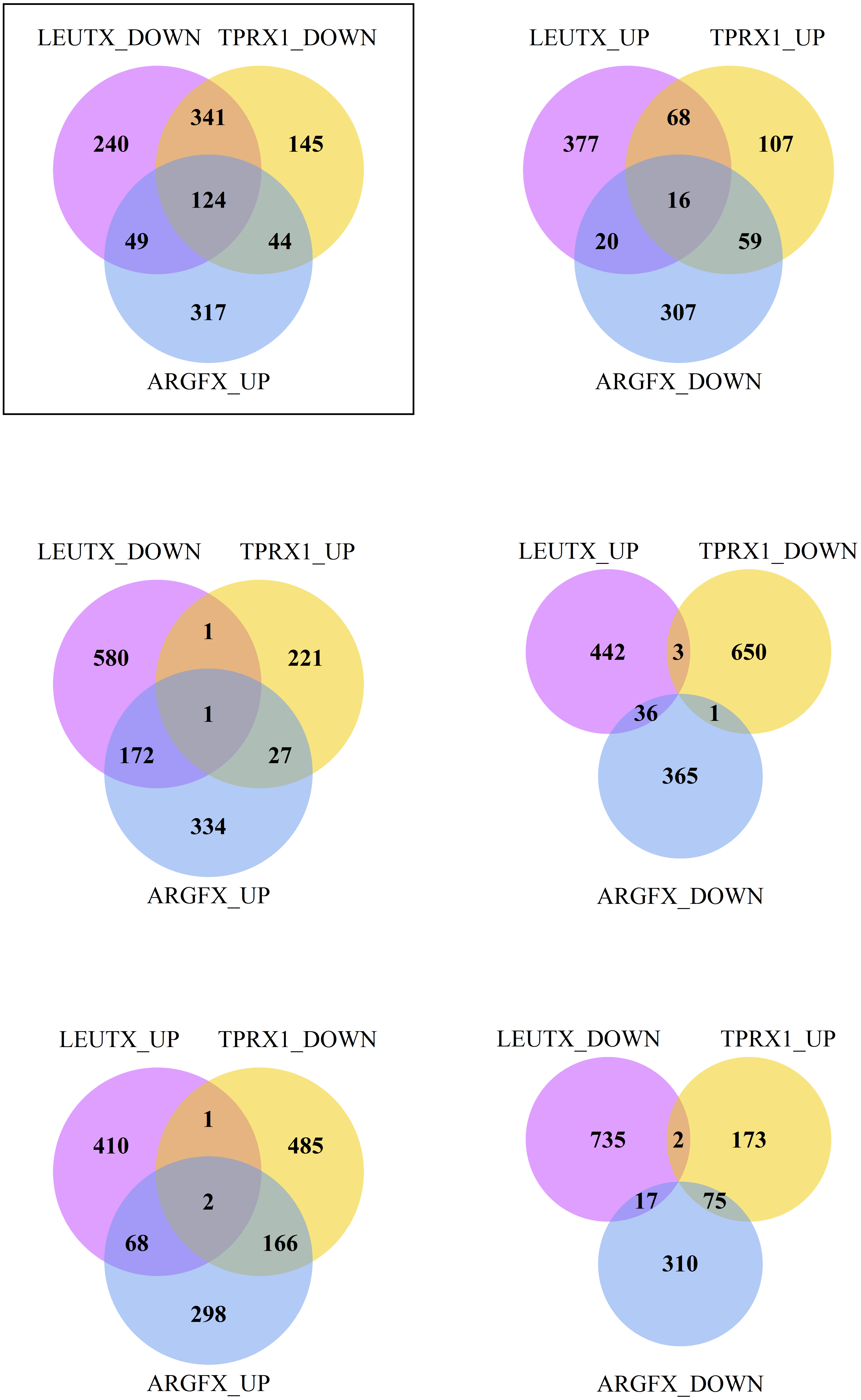

LEUTX_DOWN
TPRX1_DOWN
240
341
145
124
49
44
317
ARGFX_UP
LEUTX_UP
TPRX1_UP
377
68
107
16
20
59
307
ARGFX_DOWN
LEUTX_DOWN
TPRX1_UP
580
1
221
1
172
27
334
ARGFX_UP
LEUTX_UP
TPRX1_DOWN
442
3
650
36
1
365
ARGFX_DOWN
LEUTX_UP
TPRX1_DOWN
410
1
485
2
68
166
298
ARGFX_UP
LEUTX_DOWN
TPRX1_UP
735
2
173
17
75
310
ARGFX_DOWN
